# Physical Simulation of Phase Separation at the Slag–Metal Interface During Pellet Melting: A Phenomenological Study

**DOI:** 10.3390/ma19173779

**Published:** 2026-09-05

**Authors:** Yujian Wang, Zhuoyue Du, Guoqi Song, Lei Chen, Jie Dang, Chao Chen

**Affiliations:** 1 College of Materials Science and Engineering, Chongqing University, Chongqing 400044, China; wang2262039@126.com; 2 Panzhihua Iron & Steel Research Institute, Panzhihua 617000, China; 3 College of Materials Science and Engineering, Taiyuan University of Technology, Taiyuan 030024, China; duzhuoyue0114@link.tyut.edu.cn (Z.D.); songguoqi0443@link.tyut.edu.cn (G.S.); 4 Department of Mechanical Engineering, Taiyuan Institute of Technology, Taiyuan 030008, China; chenlei839@163.com

**Keywords:** metallized pellets, slag–metal interface, melting behavior, slag–metal separation, physical modeling, parallel flow

## Abstract

Metallized pellets are spherical iron-bearing burden materials obtained by treating iron ore pellets through processes such as direct reduction. They contain a certain proportion of metallic iron and mainly consist of metallic iron, incompletely reduced oxides, gangue, and other nonmetallic components. They are one of the commonly used iron-bearing materials in electric smelting furnaces. The melting process of metallized pellets not only affects the melting efficiency of the charge but is also accompanied by slag–metal separation and gangue separation, which is directly related to mass transfer, heat transfer, and production efficiency during the smelting process. However, existing studies have mainly focused on the melting behavior of pellets in a single-phase molten pool, while studies on gangue separation, interfacial migration, and slag–metal separation during pellet melting at the slag–metal two-phase interface remain rare. Because this region involves complex interfacial heat transfer, fluid flow, and interfacial interactions, investigating only the overall melting process of pellets is insufficient to reveal the actual gangue separation mechanism. Therefore, a systematic investigation of the melting and separation processes of pellets at the slag–metal interface is necessary. Based on the principle of similarity, a water–oil–ice three-phase physical model was employed in this study, in which water, silicone oil, and ice balls containing dyed silicone oil samples were used to simulate molten iron, slag, and pellets, respectively. The dyed silicone oil is specially designed to simulate the gangue in the pellet. Visualization experiments were conducted to investigate the evolution of pellet melting morphology, oil droplet (gangue) release behavior, and diffusion characteristics in the oil layer under different initial oil droplet positions and static or parallel flow conditions. The results show that the initial position of the oil droplet and the parallel flow significantly affect the local melting behavior of the ice ball and the oil droplet release process. In particular, the release time of the oil droplet located above the ice ball is significantly longer than that of the oil droplet located below the ice ball. The parallel flow significantly changes the melting sequence of the ice ball and the oil droplet release path by enhancing convective heat transfer in the lower region of the ice ball. According to the initial position of the oil droplet and the flow conditions, the oil droplet release process can be classified into five typical separation types, including (1) lateral release of the upper oil droplet after the ice shells on both sides melt through, (2) release of the upper oil droplet through a hole at the bottom of the upper hemisphere, (3) direct release of the lower oil droplet through a local hole, (4) two-stage release of the lower oil droplet controlled by interfacial constraint, and (5) single-stage release of the oil droplet on the downstream flow side driven by parallel flow. The oil droplet release time in the parallel flow cases is shorter than that in the static conditions. The diffusion behavior of the oil droplet after entering the oil layer is weakly affected by the parallel flow and is mainly characterized by inertial diffusion along the initial release direction, followed by spreading toward the surrounding area. This study reveals the slag–metal separation mechanism during pellet melting at the slag–metal interface under the combined control of flow, ice shell morphology, and interfacial interactions, providing experimental evidence for optimizing the melting and separation behavior of pellet charges in electric smelting furnaces and related smelting processes.

## 1. Introduction

Against the background of global responses to climate change and the advancement of the “dual carbon” goals, the steel industry, as one of the major sources of carbon emissions, is undergoing profound transformations toward low-carbon metallurgy [1,2,3,4,5]. Vanadium titanomagnetite (VTM), as a typical polymetallic symbiotic complex ore, is rich in various strategic metal resources, including iron, vanadium, and titanium [6,7,8,9,10]. For the efficient and green utilization of vanadium titanomagnetite, the short-process technology of “hydrogen-based shaft furnace direct reduction–electric smelting furnace (ESF) smelting separation” demonstrates great application potential [11,12,13,14]. In this process, vanadium titanomagnetite is reduced in a hydrogen-based shaft furnace [15,16] to obtain vanadium-titanium metallized pellets [17,18] with a high metallization degree, which are directly charged into the electric smelting furnace for melting and slag–iron separation [19,20,21]. The internal temperature of the electric smelting furnace exceeds 1733 K, and the furnace body is enclosed by opaque refractory materials [22], causing the melting of pellets at the slag–iron interface and the sedimentation separation process of slag and iron to be completely in an invisible “black box” state [21,23]. During this process, the highly metallized pellets undergo complex phase transformations and melting, where liquid iron droplets with higher density penetrate through the slag layer and settle to the bottom of the molten pool, while titanium-rich slag floats on the upper layer, thereby achieving iron–titanium separation [24,25]. However, this extreme invisibility severely restricts the in-depth understanding of the interfacial melting kinetics and the mechanism of slag–iron separation.

Currently, studies on the melting behavior of metallized pellets and slag–iron separation behavior mainly focus on three aspects: mathematical models and numerical simulations, high-temperature experiments, and physical simulations. In terms of mathematical models, Jiao and Themelis [26] established a heat transfer mathematical model for the melting of solid particles in a liquid slag or metal bath, and analyzed the influencing factors of crust formation and melting time. Subsequently, He et al. [27] developed a mathematical model for the melting of oxidized pellets in molten slag based on the one-dimensional transient heat conduction principle, revealing the specific effects of factors such as slag bath temperature, pellet metallization degree, and pellet diameter on slag crust formation and melting rate. In recent years, computational fluid dynamics (CFD) or multiphysics models have been employed by researchers to conduct numerical simulations of the pellet melting process [28,29,30,31]. For example, Lin et al. [29] and Calderon Hurtado et al. [30] utilized COMSOL Multiphysics software 6.1, and Zhu et al. [31] utilized ANSYS Fluent software, respectively, to perform numerical simulations of the melting processes of hydrogen-based direct reduced iron and metallized pellets in slag or iron/steel molten pools. However, mathematical models and numerical simulations are generally based on simplified assumptions (such as neglecting chemical reactions and treating physical property parameters as constants), making it difficult to fully reproduce the complex physicochemical and multiphase flow behaviors involved in melting and slag–iron separation.

In terms of high-temperature experiments [32,33], Shatokha and Velychko [34] elucidated the effects of basicity on the liquid phase formation temperature of pellets and slag–iron separation efficiency through high-temperature reduction experiments under load, and indicated that the metal phase and slag phase exhibited retention phenomena in the coke bed. Xing et al. [35] investigated the effects of iron bath temperature, basicity, metallization degree, and pellet-to-iron ratio on the melting rate of vanadium titanomagnetite metallized pellets. Lan et al. [36] systematically studied the effect of metallization degree on the melting behavior of hydrogen-based direct reduced iron (HDRI), and found that an increase in metallization degree gradually increased the initial melting temperature, hemispherical temperature, and slag–iron separation temperature. Pousette et al. [37] experimentally investigated the melting behavior of hydrogen-reduced pellets in electric arc furnace slag, revealing the mechanism by which slag composition controls the melting rate through its effects on thermal conductivity and convective heat transfer. Han et al. [38,39] studied the direct vortex melting reduction in vanadium-titanium magnetite on a laboratory scale and found that the vortex in the melt pool could facilitate the contact between coke and the melt. However, high-temperature experimental studies mainly focus on single molten pool environments, such as pure iron baths or pure slag baths, without considering the critical region where pellets are located at the slag–iron interface. The complex heat transfer, mass transfer, and fluid dynamic behaviors in this region have a decisive influence on the pellet melting and slag–iron separation processes.

In terms of physical models, Iwamasa and Fruehan [40] used a water–oil mixture to simulate the slag–iron mixed melt and measured the sedimentation time and coalescence critical conditions of metal droplets in the slag phase. In other research fields, Hao and Tao [41,42] analyzed the flow field characteristics around ice spheres in parallel water flow and conducted visual investigations of the mass transfer process using dyed ice spheres. In recent years, numerous studies have used ice to simulate the melting process of scrap steel [43,44,45,46,47,48]. Cao et al. [44] used ice blocks to simulate scrap steel and investigated the melting behavior of scrap steel and its influencing factors during converter steelmaking. Most studies used pure water, KCl solution [46,47,49,50], or sugar water [48] to prepare ice spheres. The ice used in these studies was a single-phase material, which cannot simulate the melting and slag–iron separation processes of pellets containing gangue. In addition, most of the aforementioned studies were conducted in static ice–water two-phase systems or ice–water–gas three-phase flow systems [46], without considering the slag phase, and most studies did not consider the influence of local flow.

In addition, under the natural convection induced by continuous feeding and electrode heating in the electric smelting furnace, significant parallel flow is often present near the slag–iron interface [51,52]. Parallel flow not only changes the melting rate of pellets but may also promote or inhibit the detachment and dispersion of droplets through shear forces, thereby significantly affecting the slag–iron separation efficiency [53,54,55]. Currently, physical simulation studies on the melting kinetics and slag–iron separation behavior of metallized pellets at the slag–iron interface under the influence of parallel flow are extremely limited.

Based on the above, this study employed a water model experiment method, in which water, silicone oil, and dyed silicone oil ice samples were used to simulate iron, slag, and pellets, respectively, thereby simulating the melting and slag–iron separation processes of metallized pellets at the slag–iron interface. The evolution of the melting morphology of metallized pellets at the slag–iron interface and the slag–iron separation phenomena were mainly investigated, and the influence laws of parallel flow on the melting rate, droplet detachment, and interfacial separation efficiency were revealed. This study provides theoretical support and data references for pellet melting and slag–iron separation in the electric smelting furnace.

## 2. Principles and Methods

### 2.1. Experimental Principle

This experiment is based on the principle of similarity and employs a physical model (water model) to investigate the melting and separation behavior of the pellets at the slag–metal interface. To ensure that the model can accurately represent the physical processes of the prototype, the experimental design must follow specific similarity criteria. In this study, similarity theoretical calculations were mainly conducted with respect to the phase-change process (melting) and interfacial properties.

(1) Geometric similarity

Details such as velocity fields are not measured in industrial ESF and are not available for model Validation. Only the pellet-scale melting and slag–metal separation processes in the local region are considered. According to the numerical simulation results of the six-electrode ESF reported by Peng et al. [23], a strong horizontal flow exists at the slag–metal interface in the ESF. Dong et al. [52] reported that the flow velocity in molten iron is 0.01–0.04 m/s. In this study, the pellet size was used as the characteristic length. An ice ball with a diameter of 50 mm was selected to simulate the pellet, while the pellet diameter was 12 mm. According to the similarity theory, the scale factor λ is defined as model: prototype = 4.17. According to the similarity of the Froude number, the flow velocity satisfies the following equation:

(1) $$\frac{v_{w}}{v_{st}}=\sqrt{\lambda }$$

where v_w_ and v_st_ are the velocities of water and steel, respectively.

At this point, the model velocity is approximately 2.04 times that of the prototype. Therefore, the velocity in the water model should be selected in the range of 0.02–0.08 m/s. As a preliminary study, a velocity of 0.03 m/s was adopted in this study.

(2) Similarity of the Phase-Change Number

The melting process of the pellets in the molten pool is mainly governed by heat transfer between the solid and liquid phases, including heat transfer between slag and pellets and between molten iron and pellets. In this experiment, the phase-change number (Ph) proposed by Shukla et al. [43] was adopted as the basis for investigating pellet melting using the water model. Because the pellets and ice have similar phase-change numbers, it is feasible to use ice balls to simulate pellets in the water model experiment.

The expression for the Ph number is as follows:

(2) $$Ph=\frac{\Delta H}{C_{ps}\cdot \Delta T}$$

where ΔH is the latent heat of melting (J/kg); C_ps_ is the specific heat capacity of the solid [J/(kg⋅K)]; and ΔT is the superheat (K). According to Equation (1), when the superheat of the ice ball is 20 K, it corresponds to a pellet superheat of 45 K.

(3) Similarity of Dynamic Viscosity

To simulate the two-phase flow and interfacial behavior between molten steel and slag during the actual smelting process, a water–oil system was employed to simulate the slag–metal system in this experiment. Therefore, the dynamic viscosity of the experimental slag–metal system needs to be consistent with that of the actual system [56,57]:

(3) $$\frac{\mu_{w}}{\mu_{st}}=\frac{\mu_{o}}{\mu_{sl}}$$

where μ_w_ is the dynamic viscosity of water; μ_o_ is the dynamic viscosity of silicone oil; μ_st_ is the dynamic viscosity of molten steel; and μ_sl_ is the dynamic viscosity of slag. The dynamic viscosity of the slag layer used in this experiment is 0.06 Pa·s [58]. The viscosity of molten iron is approximately 5.3 × 10^−3^ Pa·s. The dynamic viscosity of water at 20 °C is approximately 1 × 10^−3^ Pa·s. According to Equation (2) and the calculation, the viscosity of the silicone oil used is approximately 0.011 Pa·s.

### 2.2. Experimental Method

**Figure 1:**
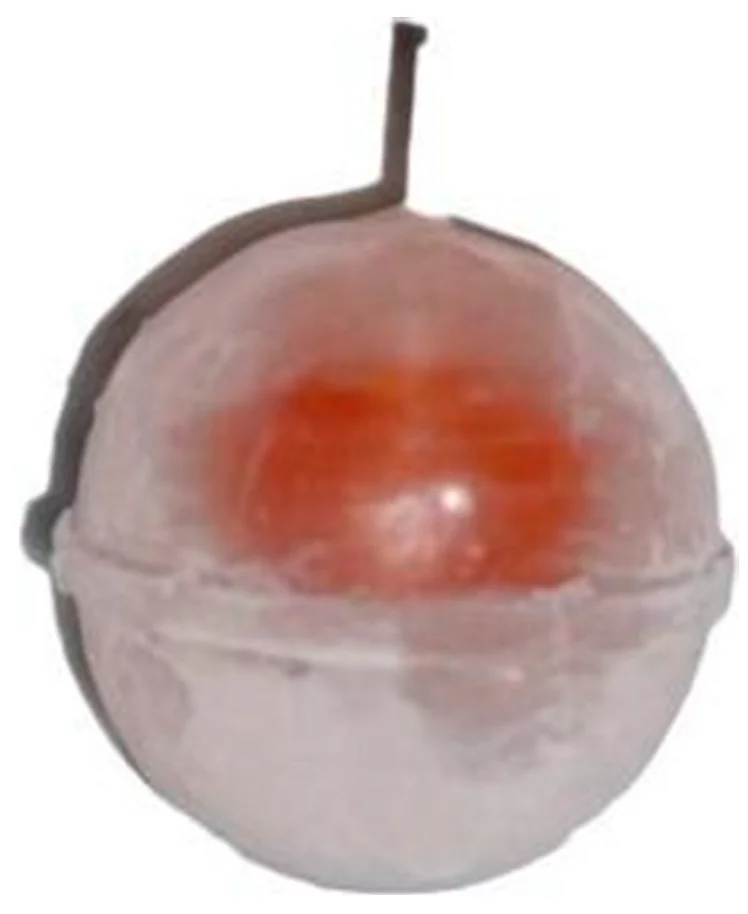
Photograph of the oil-containing ice ball.

**Figure 2:**
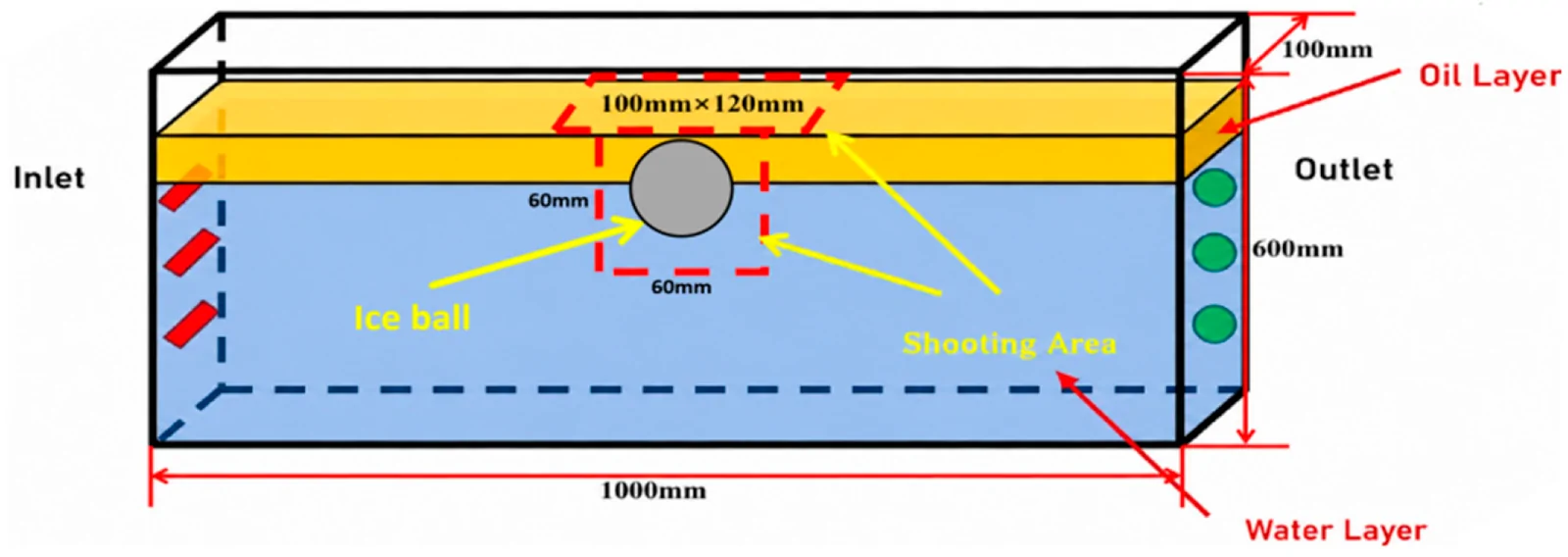
Water model experimental apparatus.

As shown in Figure 1, the ice ball used in this experiment was an oil-containing ice ball with a diameter of 50 mm and a density of 0.919 g/cm^3^, and the thickness of the oil layer was 30 mm. The overall structure and specific dimensions of the water model experimental apparatus are shown in Figure 2.

In the experiment, water was pumped from the water reservoir into the inlet on the left side of the water tank and then discharged through the outlet on the right side back into the water reservoir, thereby establishing water circulation and generating a parallel flow in the designated melting region of the water tank to meet the experimental requirements. Before each experiment, the water temperature was adjusted and stabilized at 20 °C (±0.3 °C), and the flow field was operated continuously for 10 min without the ice ball. After the flow field became stable, the ice ball was placed into the water tank to initiate the melting experiment. In this experiment, the ice ball was fixed in the imaging region shown in Figure 2, and video recording equipment was used to record the melting process of the ice ball and the specific oil droplet release process. The video recording equipment for the ice ball melting process was positioned directly in front of the imaging region, perpendicular to the wall of the water tank. In addition, for some oil droplet diffusion processes, video recording equipment positioned perpendicular to the liquid surface was used to simultaneously capture the top and front views. The preparation process of the oil-containing ice balls was as follows: water was injected into a plastic mold with a diameter of 50 mm and frozen. After a relatively thick ice shell had formed, the ice shell was pierced with a syringe, and the liquid water inside was extracted. Dyed silicone oil was then injected, after which the mold was placed in a refrigerator for storage, completing the preparation of the ice ball. To obtain ice balls with different initial oil droplet positions, the ice balls were partially melted during a secondary preparation process, and a fixed plastic rod was inserted to adjust the relative position of the oil droplet within the ice ball. After repeatedly preparing oil-containing ice balls to reduce the variation in the initial oil droplet position between experiments, ice balls with similar oil droplet positions were selected to ensure that oil-containing ice balls with the same initial state could be used in the experiments. To investigate the separation process of gangue at different positions within metallized pellets under actual production conditions, the oil droplets were placed at different positions within the ice balls, and the release behavior of the oil droplets at different positions was observed by recording the melting videos, thereby supplementing the separation phenomena that cannot be observed in actual production.

**Figure 3:**
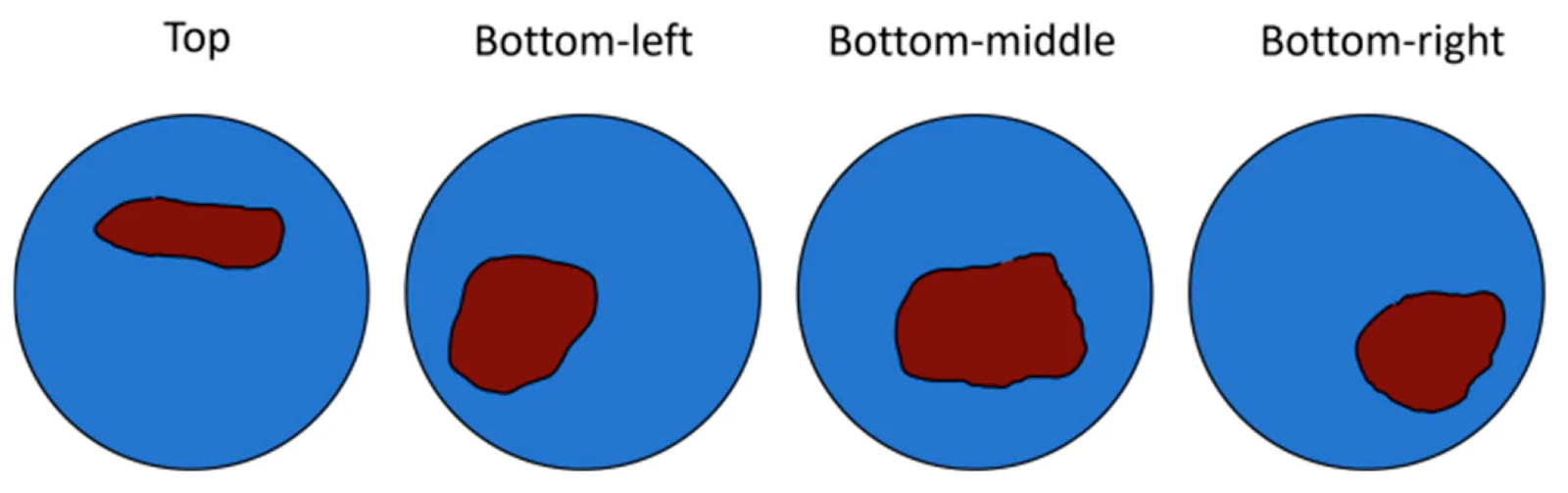
Schematic diagram of different oil droplet positions.

During the experiments, the video recording equipment continuously recorded the entire process from the initial state of the ice ball to its complete melting, with particular attention paid to the dynamic changes in the shape of the ice ball and the oil droplet release behavior under different conditions. The specific experimental conditions are listed in Table 1. The experimental conditions were divided into two groups: melting experiments under static conditions (parallel flow velocity of 0 mm/s) and melting experiments under a parallel flow velocity of 30 mm/s. Four initial oil droplet positions were set for each group, namely, above the ice ball, on the lower left side, at the lower center, and on the lower right side of the ice ball, corresponding to conditions S-1 to S-4 for the static group and F-1 to F-4 for the flow group, respectively. The numbering rules were consistent between the two groups. In all experiments, the ice ball was immersed with 1/2 in the oil layer and 1/2 in the water layer, and the melting environment temperature was maintained at 20 °C. The four different positions of the oil droplet within the oil-containing ice ball are shown in Figure 3.

## 3. Results and Discussion

### 3.1. Melting Behavior of the Ice Ball Under Static Conditions

#### 3.1.1. Melting of the Ice Ball Under the S-1 Condition

**Figure 4:**
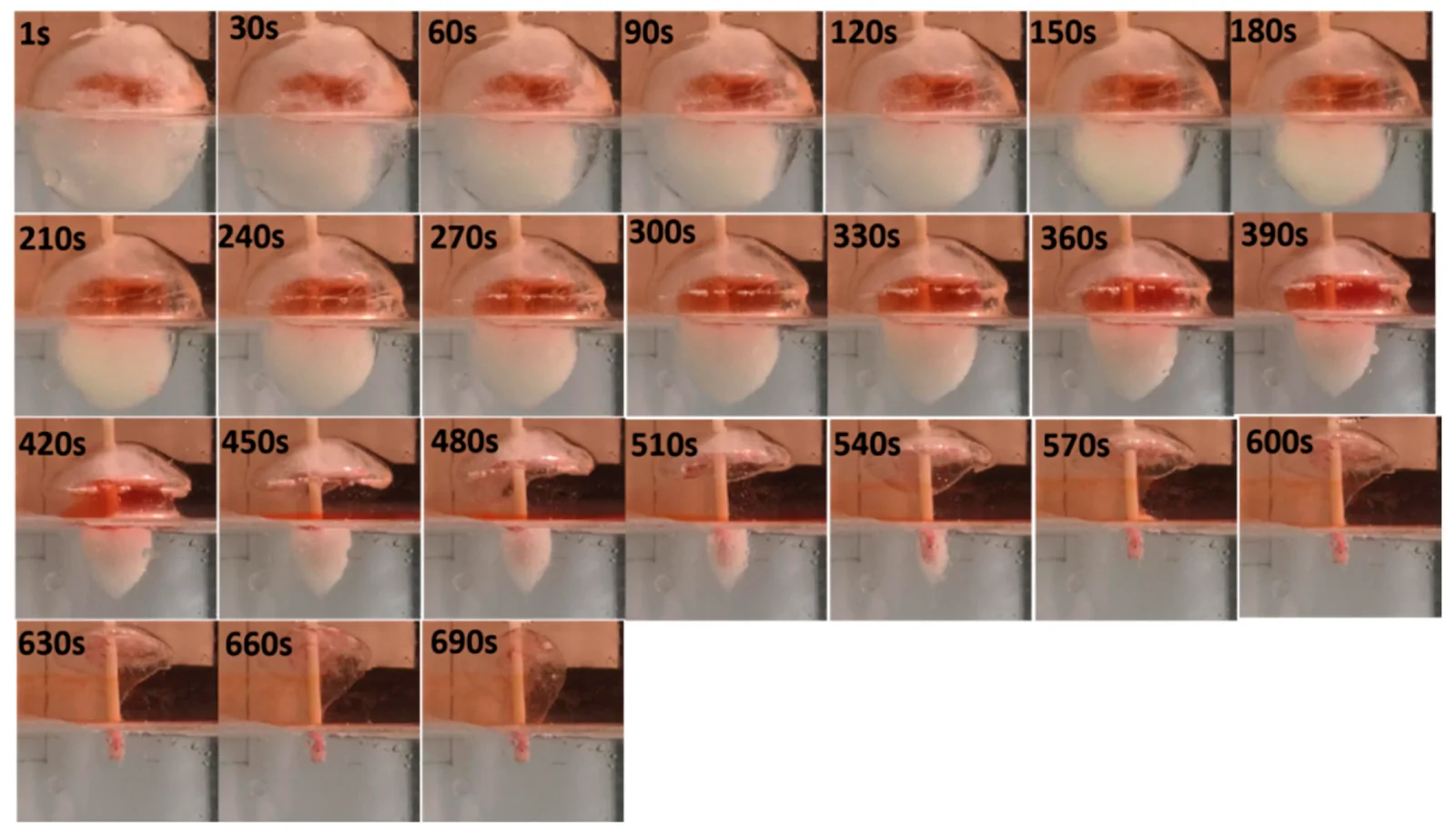
Melting process of the ice ball under the S-1 condition.

The first static condition is that the oil droplet is located in the region above the ice ball. The overall melting process of the ice ball is shown in Figure 4. During the initial 60 s, the overall morphology of the ice ball changes only slightly. It can be observed that the size of the ice ball in water is slightly smaller than that in oil. As time progresses, the portion of the ice ball immersed in water melts relatively rapidly and gradually changes from a spherical shape to an ellipsoidal shape. The portion of the ice ball immersed in the oil layer maintains a hemispherical profile throughout the melting process and uniformly shrinks inward. The upper ice layer gradually shrinks and becomes thinner in a slow and uniform manner, maintaining its hemispherical shape even at 210 s, whereas the lower portion has gradually evolved from its initial shape into a semi-ellipsoidal morphology by 240 s. Between 210 and 420 s, the outer surface of the ice layer enclosing the oil layer above the ice ball exhibits a concave morphology toward the interior of the ice ball, and local melting accelerates. At 419 s, the ice shell surrounding the oil droplet in the upper part of the ice ball has completely melted, and the oil droplet begins to flow outward. By 422 s, the oil droplet has completely merged into the oil layer. At this point, the upper and lower parts of the ice body are completely separated: the residual ice body in the upper part forms a cap-shaped structure, while the lower part forms a conical ice structure. The white part in the figure is the fixed plastic rod. At 570 s, the lower ice cone has completely melted, whereas the upper cap-shaped structure does not completely melt until 690 s, resulting in a difference of approximately 120 s between the complete melting times of the two parts. It should be noted that after 420 s, the ice block on the plastic rod continues to melt, which differs from the actual situation. Under actual conditions, the ice ball would separate into two parts, each of which would melt at the oil–water interface.

**Figure 5:**
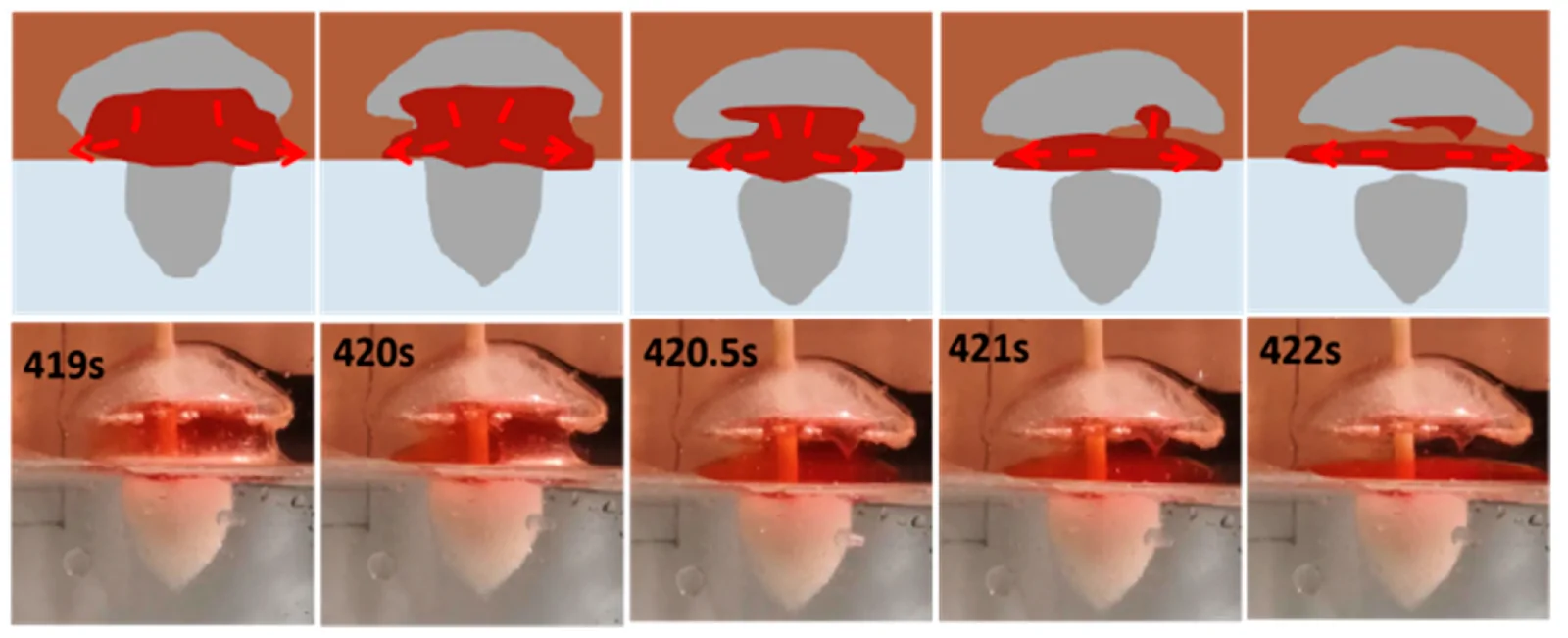
Front view of oil droplet release process under the S-1 condition.

**Figure 6:**
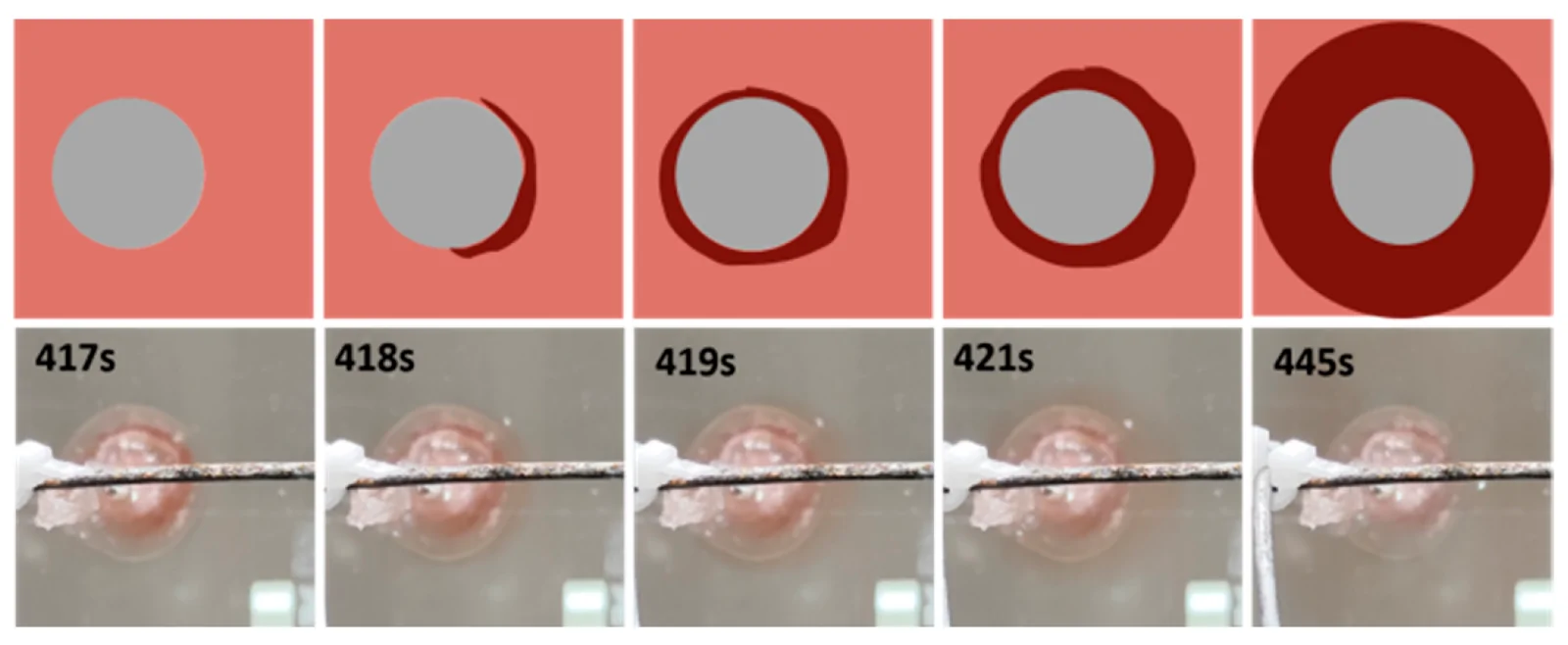
Top view of oil droplet diffusion process under the S-1 condition.

As shown in Figure 5, the oil droplet release process from the oil-containing ice ball under the S-1 condition occurs between 419 and 422 s. When the ice shell surrounding the oil droplet has completely melted on both sides, the oil droplet is immediately exposed to the surrounding oil layer. At 1 s after the oil droplet is exposed, it slides toward the oil–water interface, while its thickness gradually decreases during this process. At the second, the oil droplet completely separates from the residual ice body above, and this separation process occurs rapidly. After separation, the oil droplet immediately diffuses and spreads outward into the oil layer outside the ice shell. From the top view in Figure 6, it can be clearly observed that the oil droplet spreads uniformly during the diffusion process, gradually forming a circular oil film region centered on the ice ball. At 4 s after exposure, the spreading range of the oil droplet exceeds the region occupied by the ice ball. At 24 s after the oil droplet leaks out, the radius of the circular region formed by the dyed oil droplet in the oil layer has reached twice the radius of the ice ball, forming a standard circular region.

#### 3.1.2. Melting of the Ice Ball Under the S-2 Condition

**Figure 7:**
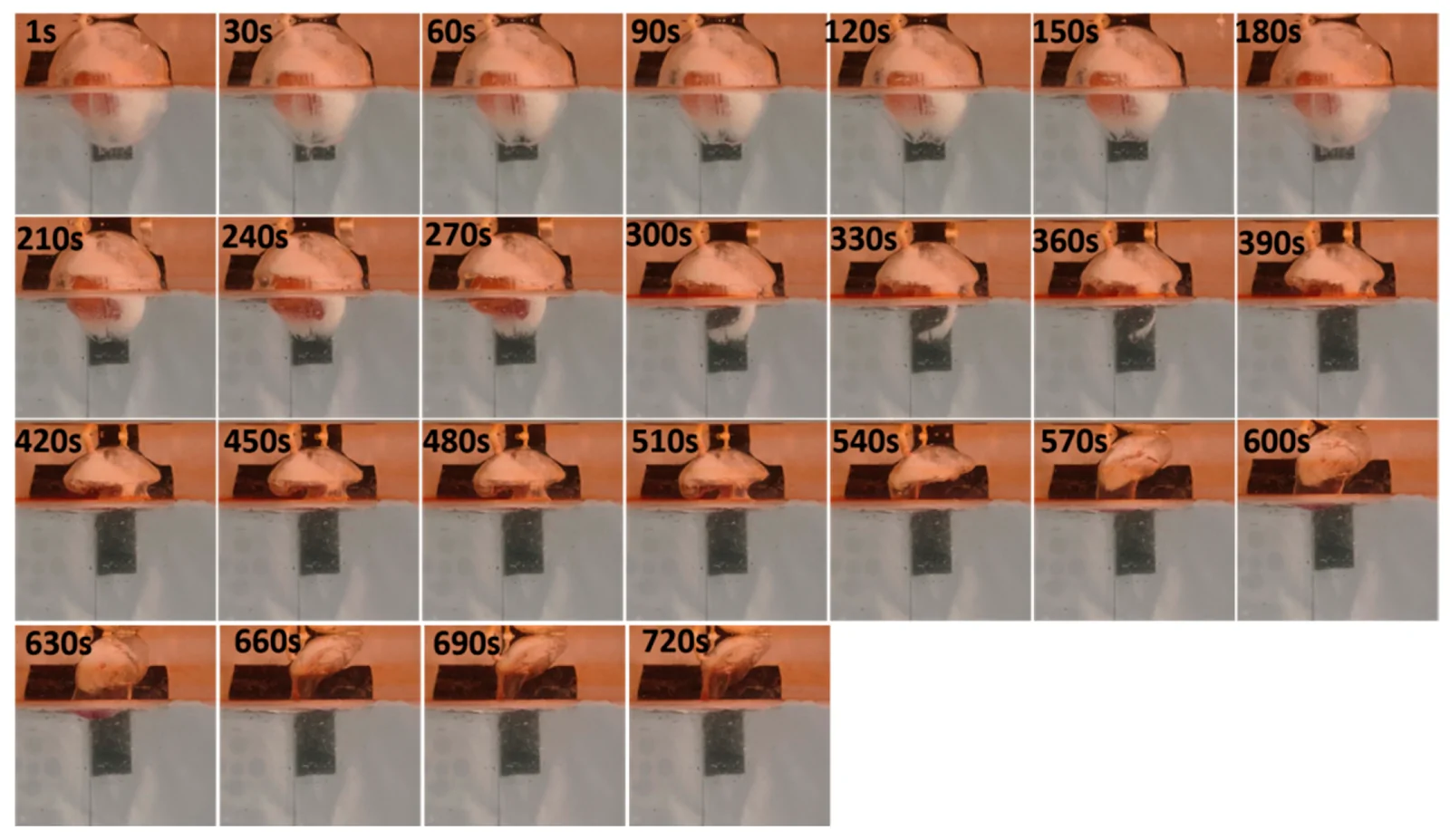
Melting process of the ice ball under the S-2 condition.

As shown in Figure 7, the upper part of the ice ball undergoes only slight changes during the initial stage of melting and generally exhibits a characteristic of uniform hemispherical shrinkage and melting. In contrast, before the oil droplet flows out, the lower part of the ice ball gradually changes from a circular shape to an ellipsoidal shape, similar to that under the S-1 condition, and melts at a relatively high rate. At 272 s, the oil droplet is exposed to the water and begins to rise toward the oil layer, where it starts to diffuse. The oil droplet rapidly flows out of the ice ball, and at 275 s, the oil droplet has completely merged into the oil layer. It is noteworthy that when the oil droplet has completely merged into the oil layer, a portion of ice remains on the right side of its original position and has not completely melted. Unlike the S-1 condition, the ice ball above and below the oil droplet does not separate. In addition, because the initial position of the oil droplet is biased toward the lower part, the melting of the lower part of the ice ball exhibits a pronounced asymmetric morphology. At 369 s, the portion of the ice ball immersed in water has completely melted, whereas the upper part of the ice ball has melted by only approximately 20% relative to its initial morphology. The time required for the complete melting of the upper part of the ice ball is approximately twice that required for the melting of the lower part, and it does not completely melt until 740 s.

**Figure 8:**
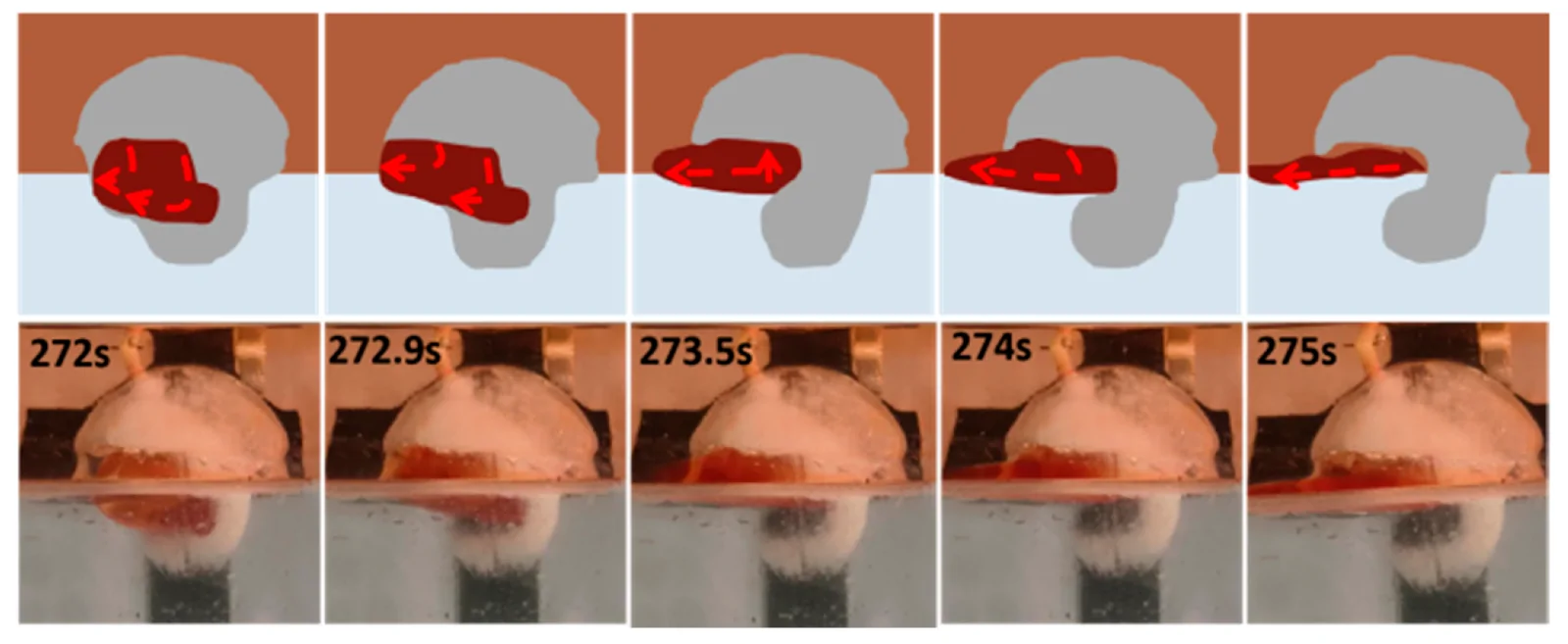
Front view of oil droplet release process under the S-2 condition.

**Figure 9:**
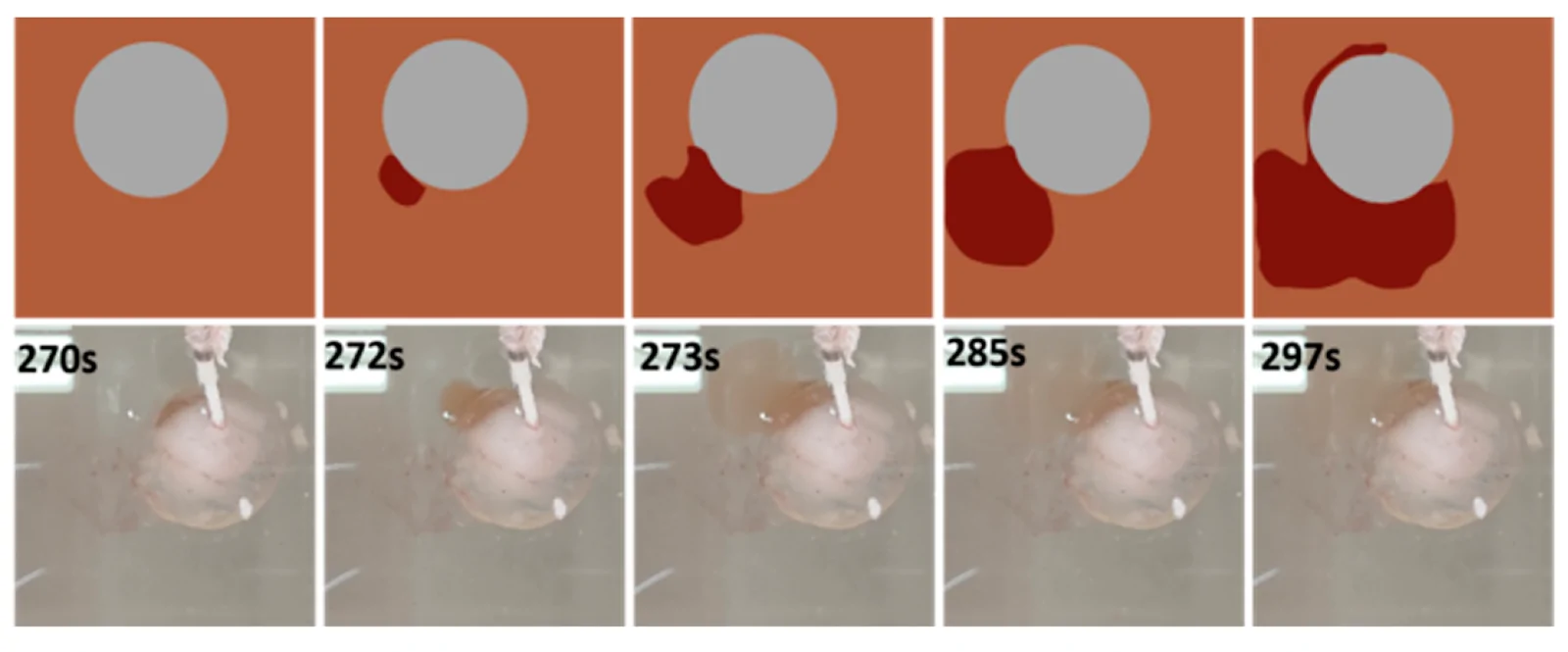
Top view of oil droplet diffusion process under the S-2 condition.

As shown in Figure 8, the oil droplet release process under the S-2 condition occurs with the ice shell melting at the same rate on both sides. The outer shell of the ice ball gradually becomes thinner, resulting in the formation of a hole in the ice ball. At 272 s, the oil droplet first begins to flow outward through the hole on the left side. Between 272 and 273 s, the oil droplet moves toward the hole on the left side, while the oil droplet below the oil–water interface completely rises and merges into the oil layer, leaving only a small portion of the oil droplet remaining inside the ice shell above the interface. Subsequently, this small portion of the oil droplet gradually begins to descend toward the hole on the left side of the ice ball and flows out between 273 and 274 s. At 275 s, the oil droplet completely separates from the upper ice ball and spreads out in the oil layer. From the top view of the oil droplet diffusion process shown in Figure 9, it can be observed that after flowing into the oil layer, the oil droplet first diffuses for a certain distance in the direction of outflow under the influence of inertia, after which the outflowing oil droplet spreads outward in all directions.

#### 3.1.3. Melting of the Ice Ball Under the S-3 Condition

**Figure 10:**
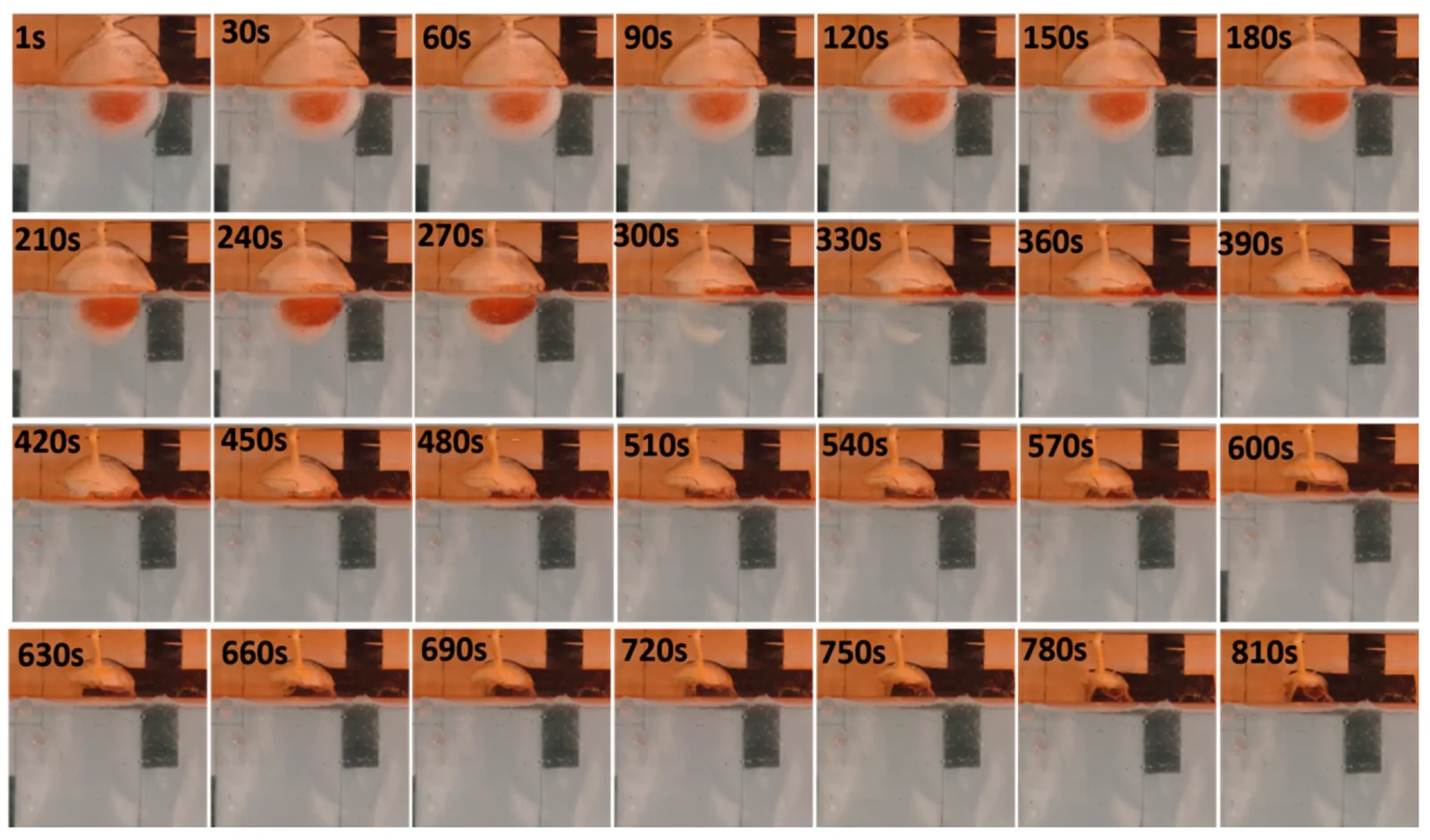
Melting process of the ice ball under the S-3 condition.

As shown in Figure 10, when the oil droplet is located in the middle of the lower part, its melting process is similar to that under the previous S-2 condition. The melting of the upper part exhibits a slow hemispherical melting behavior, whereas the melting of the lower part is characterized by simultaneous thinning on both sides. At 271 s, the oil droplet begins to flow outward, and after 5 s, the oil droplet has completely merged with the oil layer. Due to the initial position of the oil droplet, the lower part also exhibits an irregular ice shell after the oil droplet flows out. At 352 s, the lower ice ball has completely melted, whereas the ice ball in the upper oil layer does not completely melt until 822 s.

**Figure 11:**
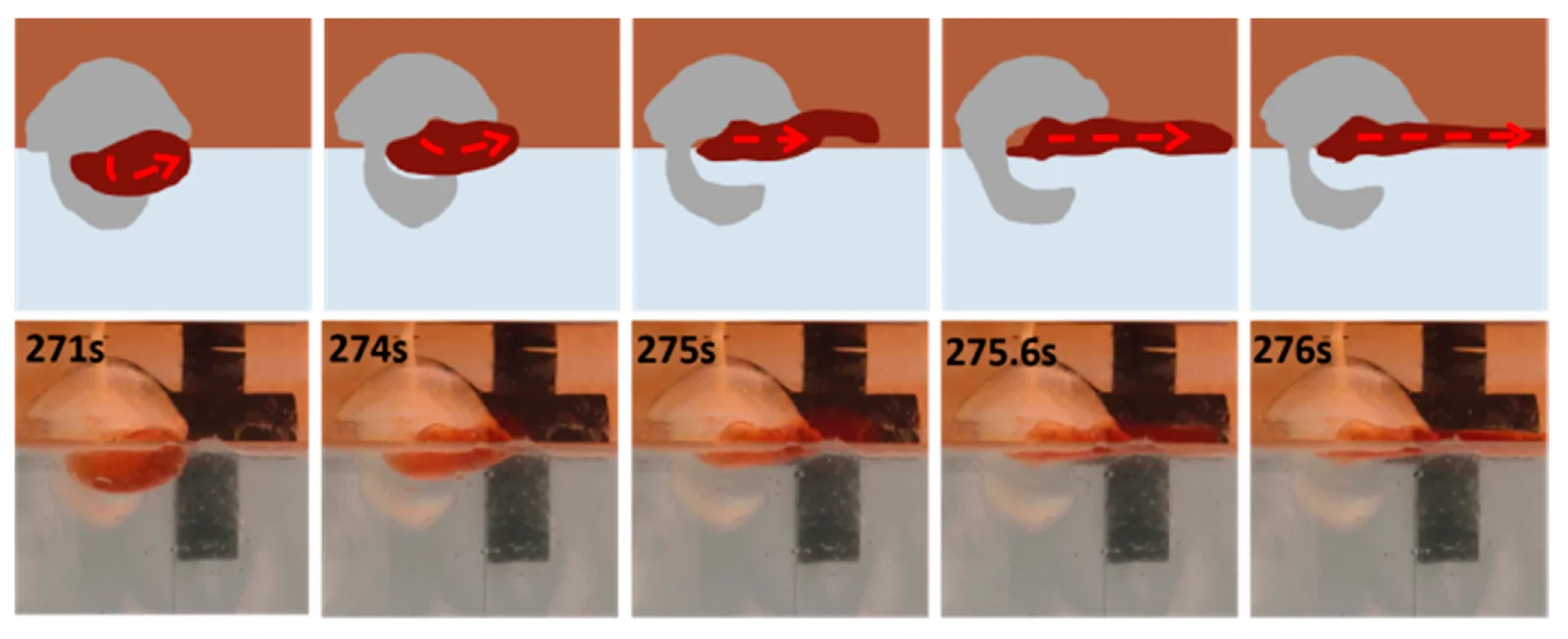
Front view of oil droplet release process under the S-3 condition.

**Figure 12:**
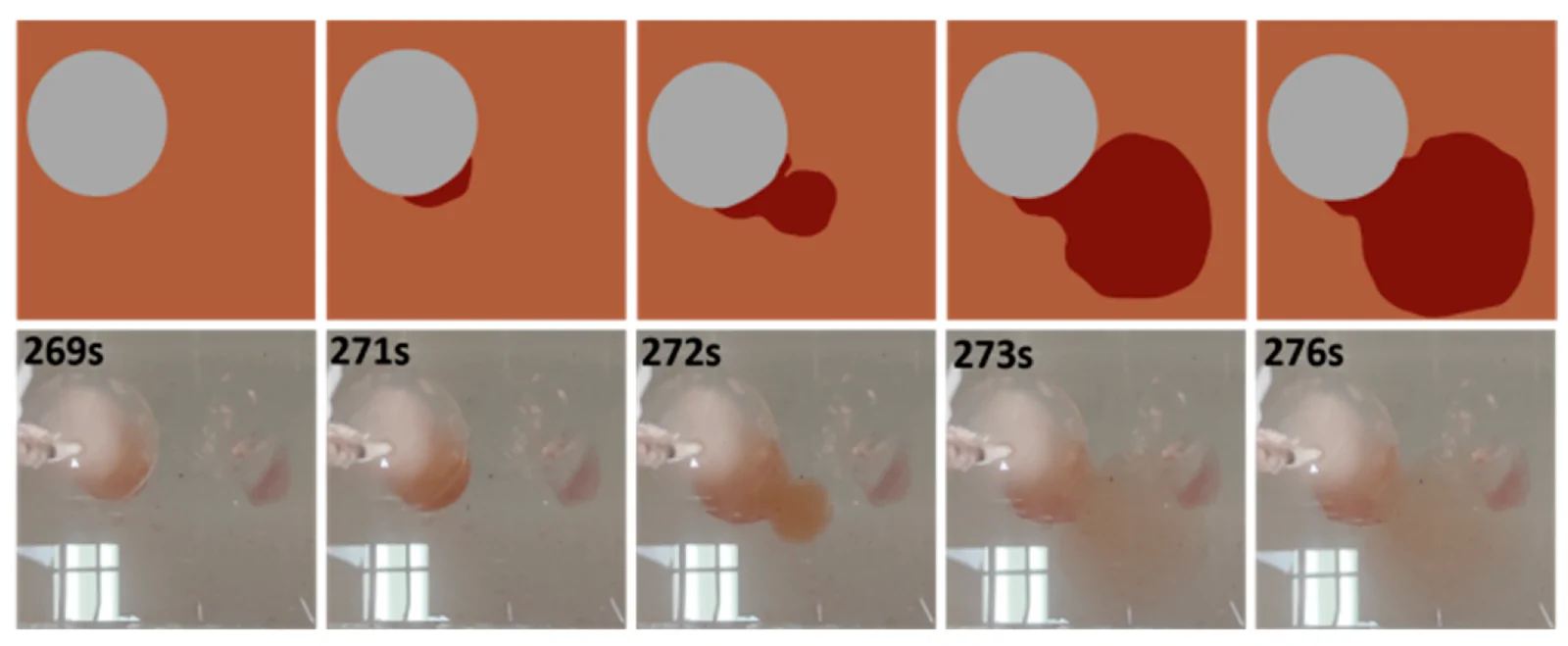
Top view of oil droplet diffusion process under the S-3 condition.

As shown in Figure 11, the oil droplet release process is related to the melting of the ice shells on both sides. When the oil droplet is located in the middle of the lower part, the ice shells on both sides of the melting oil droplet below the ice ball gradually become thinner simultaneously. A hole first forms on the right side. At 271 s, the hole is formed, while the left side is blocked by the ice ball, causing the oil droplet to slowly diffuse toward the right side and merge into the oil layer. Three seconds after the hole forms, the oil droplet below the oil–water interface separates from the lower ice shell and rapidly rises under the action of buoyancy. At 276 s, the oil droplet has completely merged into the oil layer. From the top view in Figure 12, it can be observed that after flowing out, the oil droplet first diffuses for a certain distance in the direction of outflow. Subsequently, due to the adhesion of the oil layer, the outflow velocity decreases, after which the oil droplet spreads outward in all directions. This is similar to the oil droplet diffusion process under the S-2 condition.

#### 3.1.4. Melting of the Ice Ball Under the S-4 Condition

**Figure 13:**
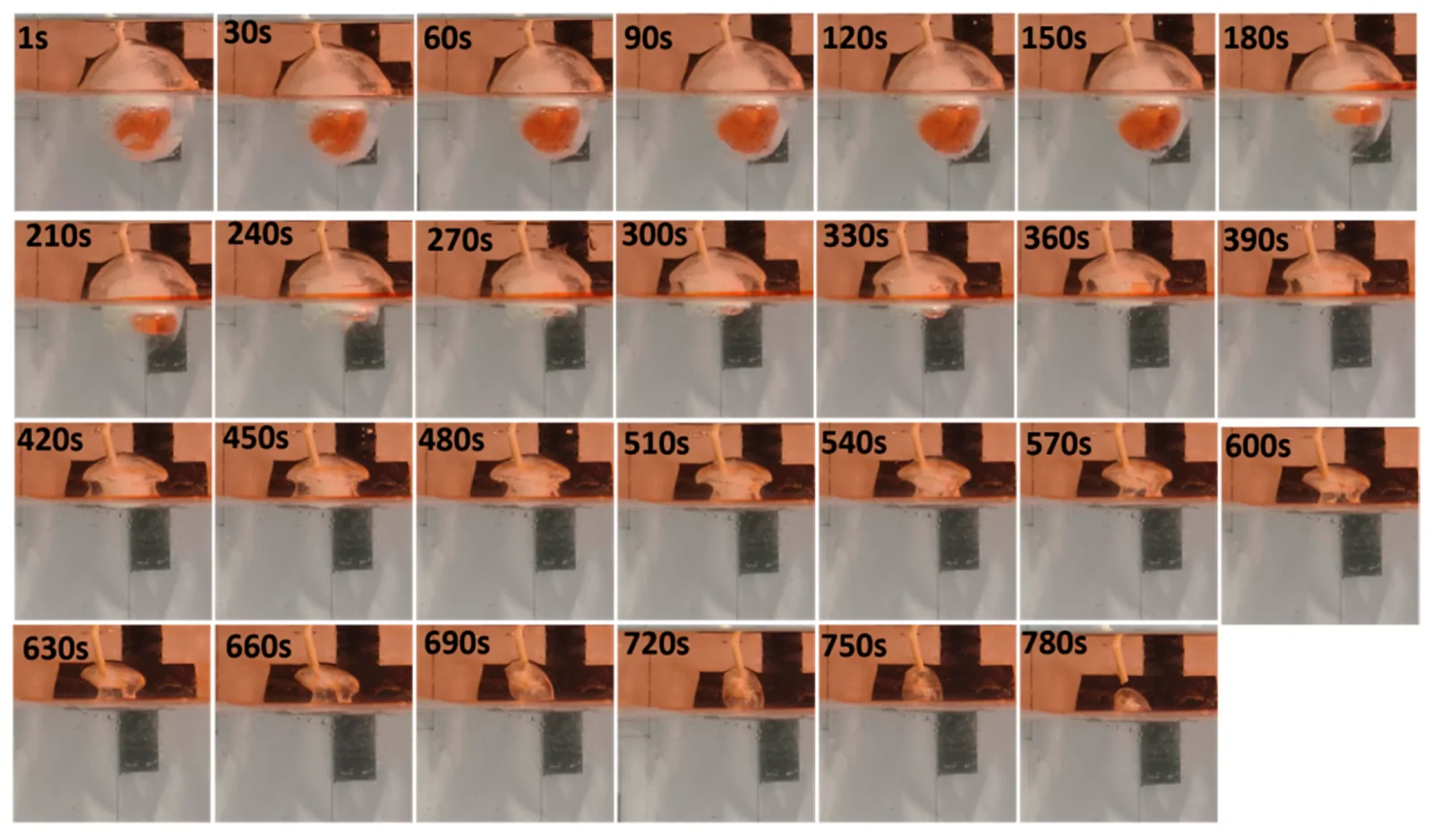
Melting process of the ice ball under the S-4 condition.

As shown in Figure 13, when the oil droplet is located in the lower right part, the melting process of the ice ball is similar to that under the previous conditions. The lower part also undergoes simultaneous thinning on both sides until the oil droplet flows out. At 174 s, a hole first appears in the ice ball at a position below the oil droplet, causing most of the oil droplet to flow out at 179 s. The early release of the oil droplet leaves a cavity at the original oil-containing position of the ice ball. Water occupies the original position of the oil droplet, resulting in accelerated melting at the bottommost part. The melting of the lower part of the ice ball exhibits a pit-like morphology, in which the bottommost part completely melts while both sides simultaneously become thinner. After a further 46 s, the lower pit expands further, and the remaining oil droplet begins to flow outward at 225 s and completely flows into the oil layer after 5 s. At 425 s, the lower ice ball has completely melted, whereas the upper ice ball does not completely melt until 807 s.

**Figure 14:**
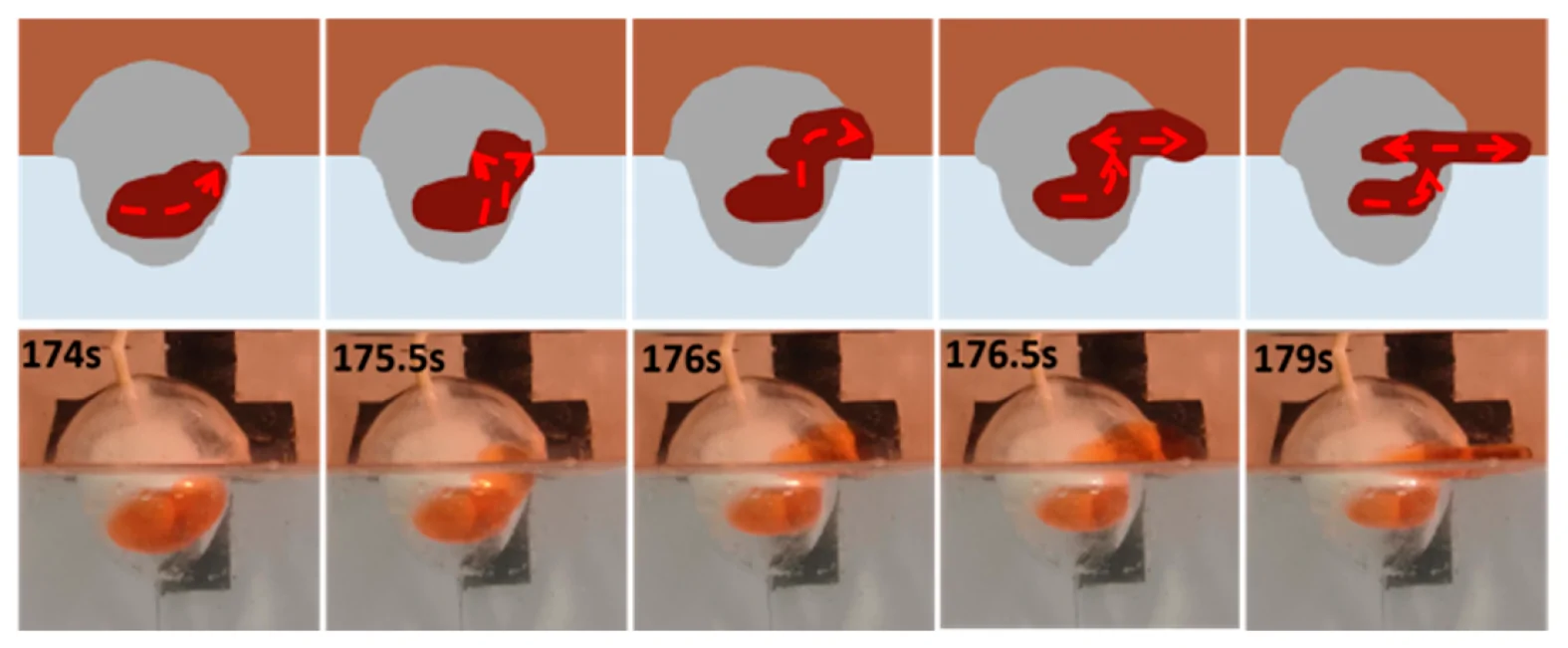
Front view of oil droplet release process under the S-4 condition (first release).

**Figure 15:**
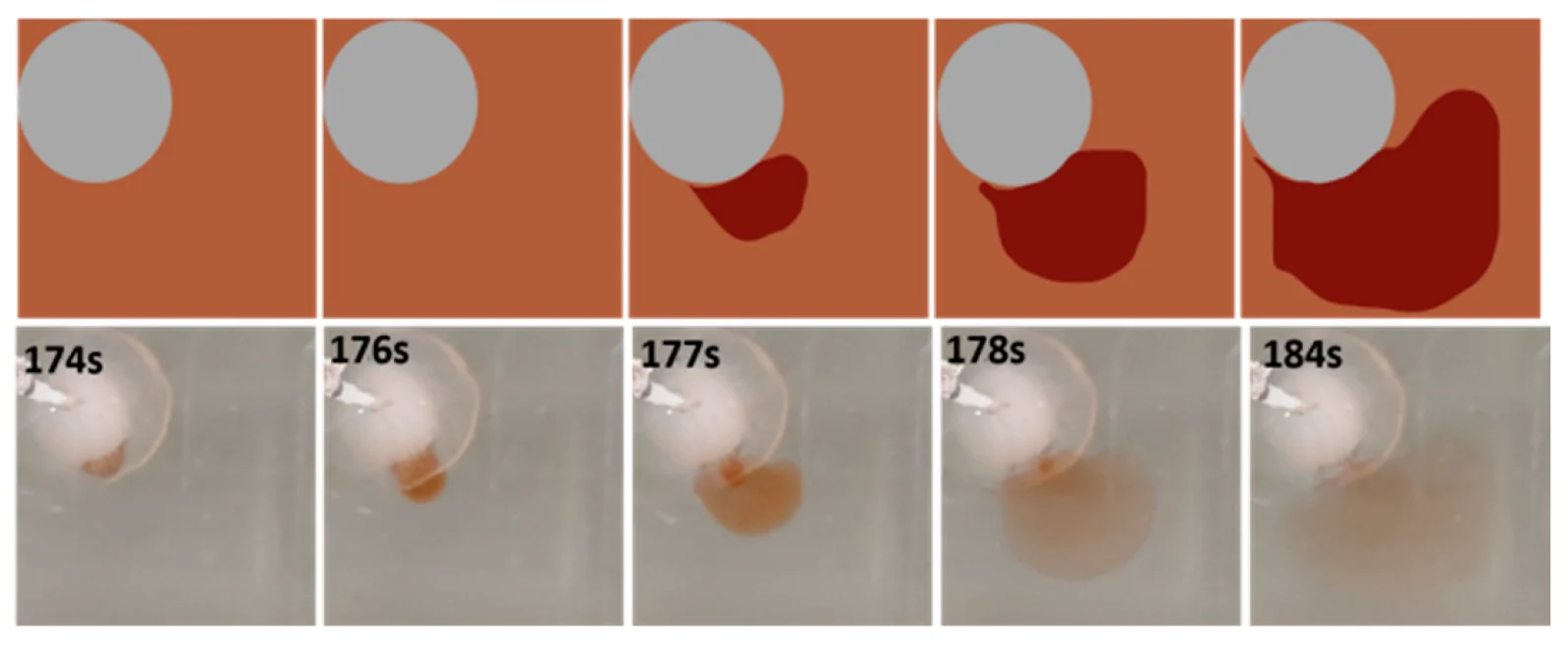
Top view of oil droplet diffusion process under the S-4 condition (first release).

**Figure 16:**
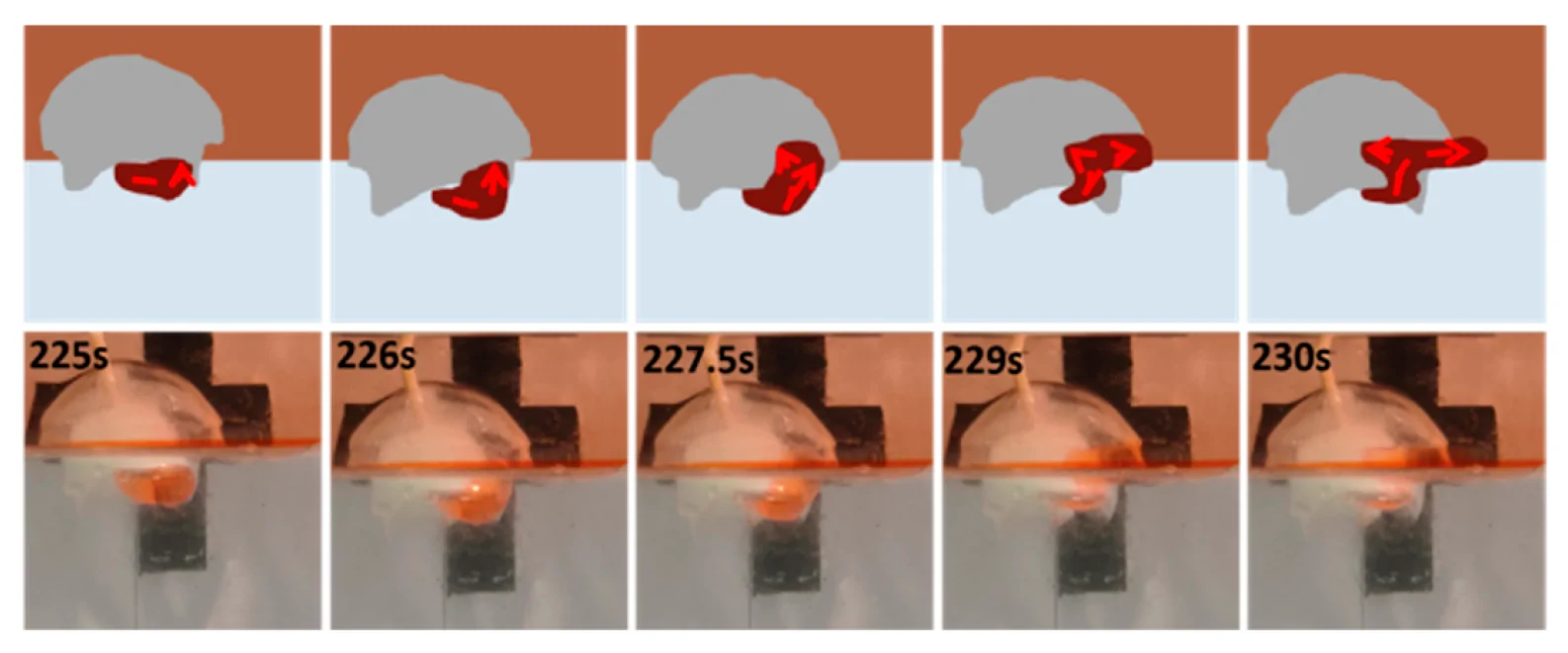
Front view of oil droplet release process under the S-4 condition (second release).

**Figure 17:**
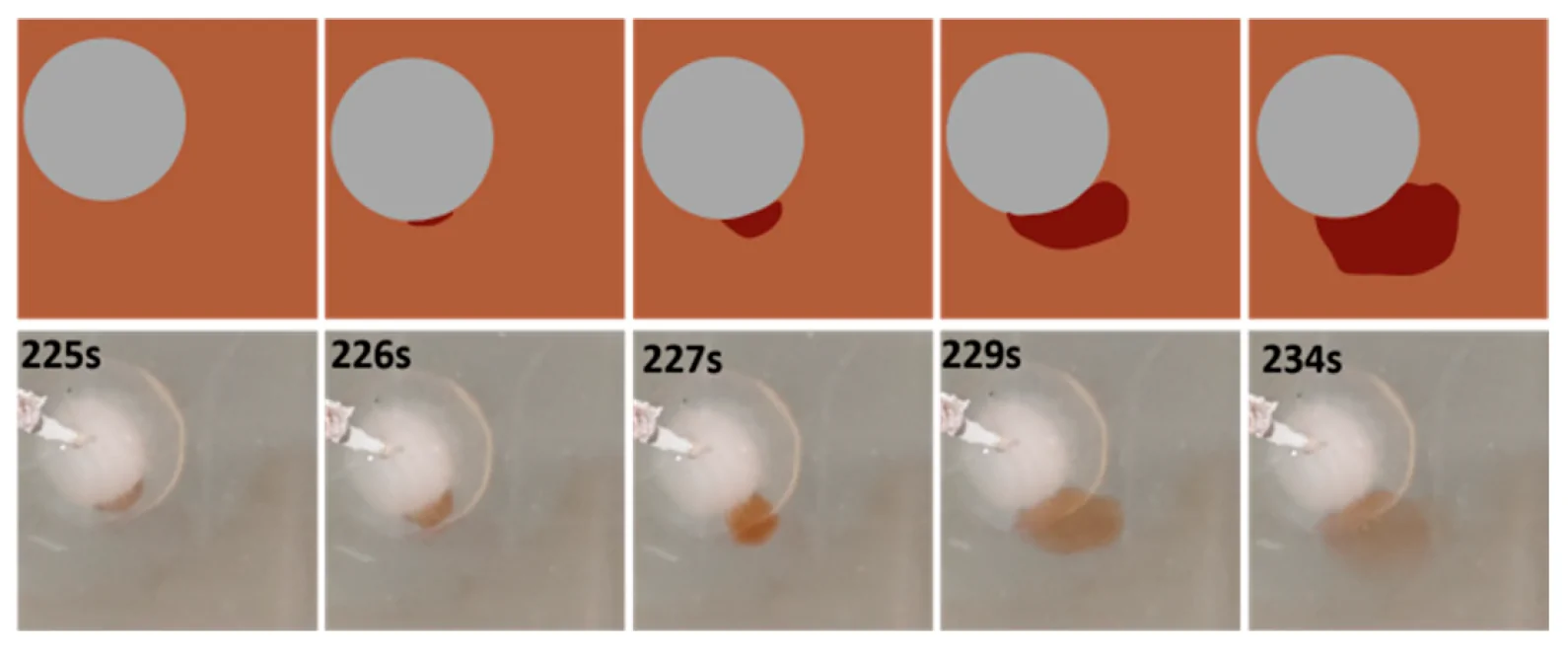
Top view of oil droplet diffusion process under the S-4 condition (second release).

As shown in Figure 14, Figure 15 and Figure 16, under the S-4 condition, the oil droplet exhibits two separate release events. Melting first forms a hole in the lower right part of the ice ball, causing the oil droplet to become exposed and flow out. Because the wall thickness of the ice layer around the oil layer is relatively large, a portion of the oil droplet flows out through the initially formed hole before the ice layer on the right side has completely melted, and then rises into the oil layer. At this point, in the absence of parallel flow, the oil droplet is affected only by buoyancy and gravity, rising vertically into the oil layer region above the hole and then spreading toward both sides, leaving a small amount of oil droplet at the center of the ice ball. As melting proceeds, the initially formed hole gradually expands and connects with the oil layer. The oil droplet subsequently rises and slides into the oil layer, spreading toward both sides and merging into the surrounding oil layer. As shown in Figure 15 and Figure 17, the positions of the two oil droplet release events are the same. After flowing out of the ice ball, the oil droplet exhibits the same phenomenon as previously observed, first flowing outward for a certain distance and then spreading outward in the surrounding oil layer.

### 3.2. Melting Behavior of the Ice Ball Under Parallel Flow

#### 3.2.1. Melting of the Ice Ball Under the F-1 Condition

**Figure 18:**
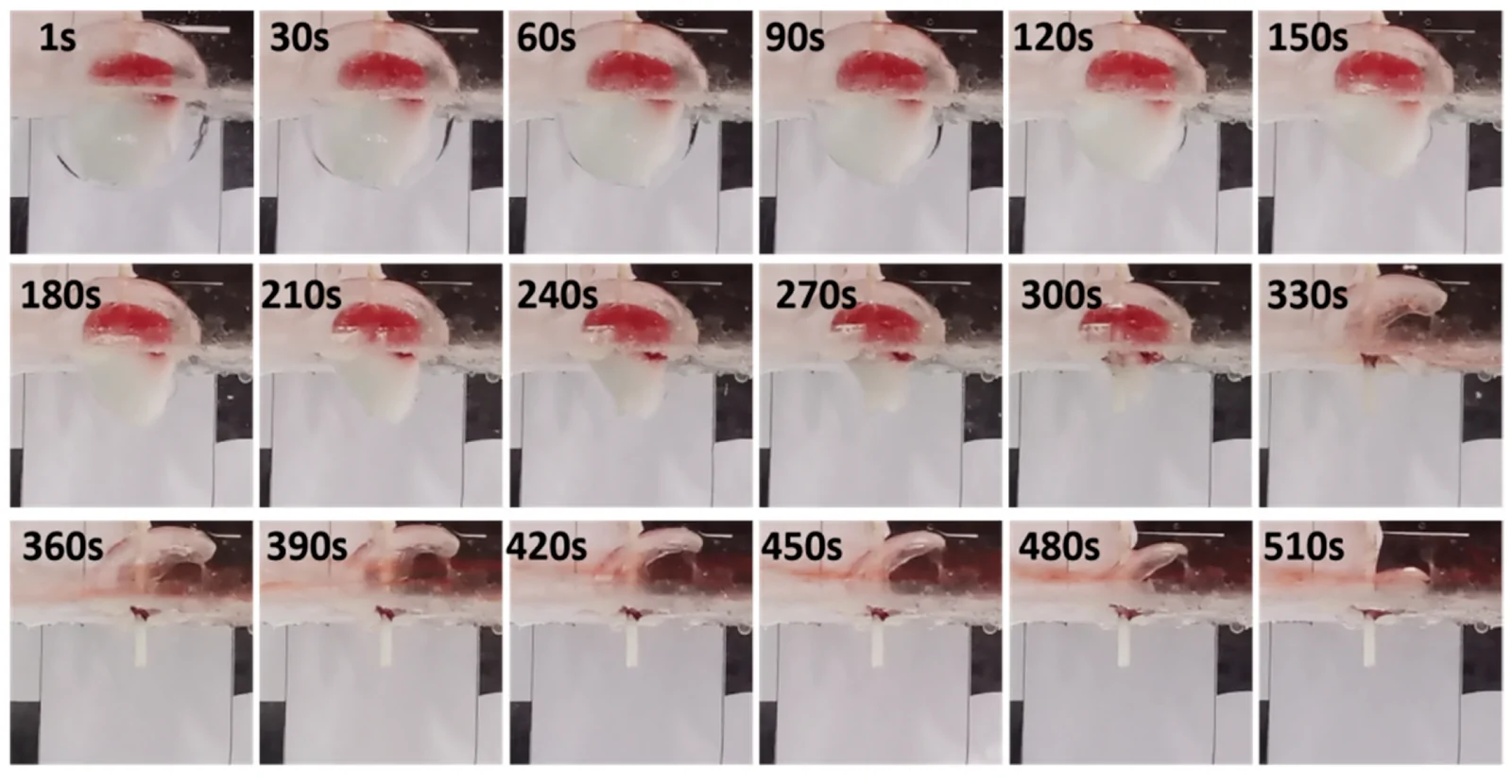
Melting process of the ice ball under the F-1 condition.

As shown in Figure 18, the upper part of the ice ball immersed in the oil layer does not differ significantly from that under the S-series conditions and maintains a hemispherical profile with slow melting. Between 90 and 270 s, as time progresses, the melting volume on the left side of the lower ice ball gradually becomes greater than that on the right side, and its morphology gradually changes from a semi-ellipsoidal shape to a melting morphology resembling an inverted triangle. This indicates that the melting on the left side is faster than that on the right side, which is significantly different from the S-1 condition. The convective heat transfer induced by the parallel flow changes the melting process of the ice ball. At 310 s, the lower ice layer has completely melted, and a small hole forms in the ice layer directly below the oil droplet. Subsequently, at 313 s, the oil droplet begins to flow outward through the hole directly below the oil droplet, and after 6 s, the oil droplet has completely merged into the oil layer. After the oil droplet has completely flowed out, the ice shell melts and breaks apart after another 10 s, changing from the originally relatively regular hemispherical shell shape into an irregular damaged shell structure. The damaged ice shell then completely melts at 514 s.

**Figure 19:**
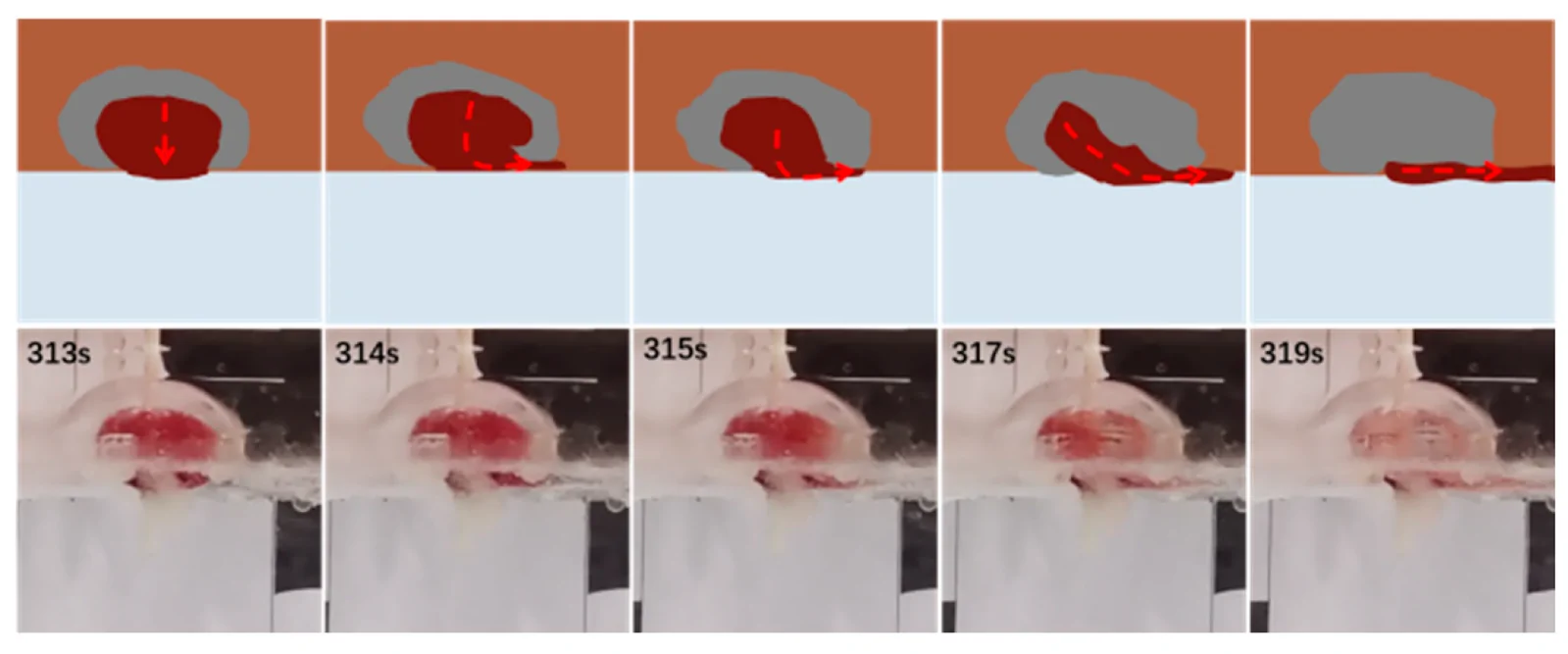
Front view of oil droplet release process under the F-1 condition.

As shown in Figure 19, the oil droplet release process occurs after the lower part of the ice ball has completely melted under the erosion of the parallel flow. Because the ice shell below the oil droplet is relatively thin, it melts through first, forming a hole. At 313 s, the oil droplet begins to flow outward through the hole and merge into the oil layer. During the first 1 s of outflow, the oil droplet flows toward the right side along the hole. Between 1 and 3 s, the oil droplet inside the ice ball gradually sinks downward and approaches the hole, continuously flowing outward. At 6 s, the interior of the ice ball becomes completely transparent, indicating that the oil droplet has completely flowed out. The initial conditions of the ice ball under F-1 are the same as those under S-1; however, due to the influence of the parallel flow, different phenomena from those under S-1 are observed. Because the upper ice shell is less affected by the flow, its melting rate is significantly slower. Therefore, when the lower ice body has completely melted and disappeared, the upper ice shell can still completely enclose the oil droplet. This is in clear contrast to the corresponding static condition. Under S-1, the ice shells on both sides of the oil droplet almost simultaneously melt through, causing the oil droplet to flow out laterally before the lower ice has completely melted, thereby resulting in the separation of the upper and lower parts of the ice ball. Under the F-1 condition, the erosion of the parallel flow enhances convective heat transfer in the lower part, causing the lower ice ball to melt completely at an earlier stage. Consequently, the oil droplet flows downward through a hole at the bottom.

#### 3.2.2. Melting of the Ice Ball Under the F-2 Condition

**Figure 20:**
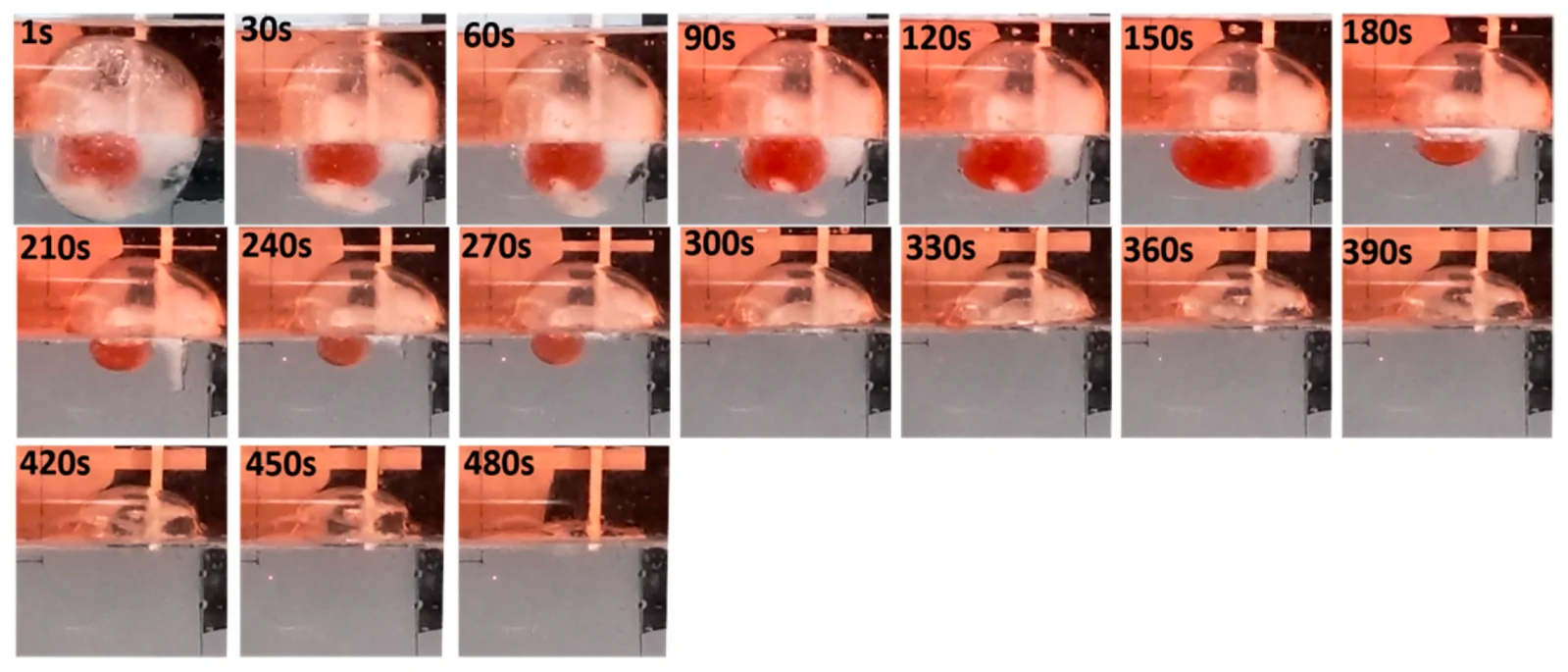
Melting process of the ice ball under the F-2 condition.

As shown in Figure 20, the oil droplet is located in the lower left region of the ice ball. During the initial 60 s of melting, the lower left region of the ice ball rapidly becomes thinner and quickly changes from a hemispherical shape to an inverted-triangular morphology. At 90 s, a hole forms in the melting ice ball, exposing a small portion of the oil droplet to the water; however, the oil droplet does not flow directly out through the hole. Between 90 and 141 s, the hole gradually expands, and the oil droplet begins to flow outward at 142 s. At 158 s, a relatively large portion of the oil droplet separates and flows into the oil layer first, leaving a small portion of the oil droplet at its original position. At 251 s, the lower ice layer has completely melted, but this small portion of the oil droplet remains at its original position and does not slide into the oil layer. Subsequently, the upper ice ball gradually melts and decreases in volume. At 276 s, the oil droplet slides into the oil layer. Subsequently, the volume of the upper ice ball gradually decreases. At 468 s, the ice ball detaches from the fixed rod and sinks downward to the oil–water interface, and completely melts at 483 s.

**Figure 21:**
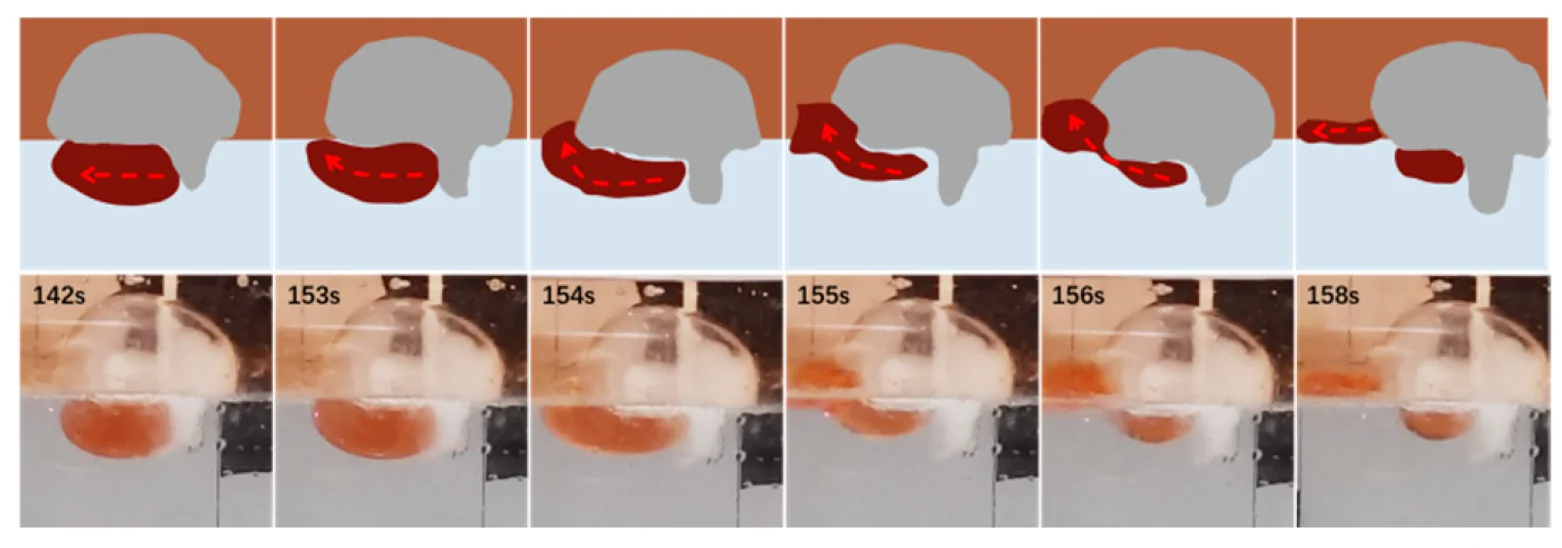
Front view of oil droplet release process under the F-2 condition (first release).

**Figure 22:**
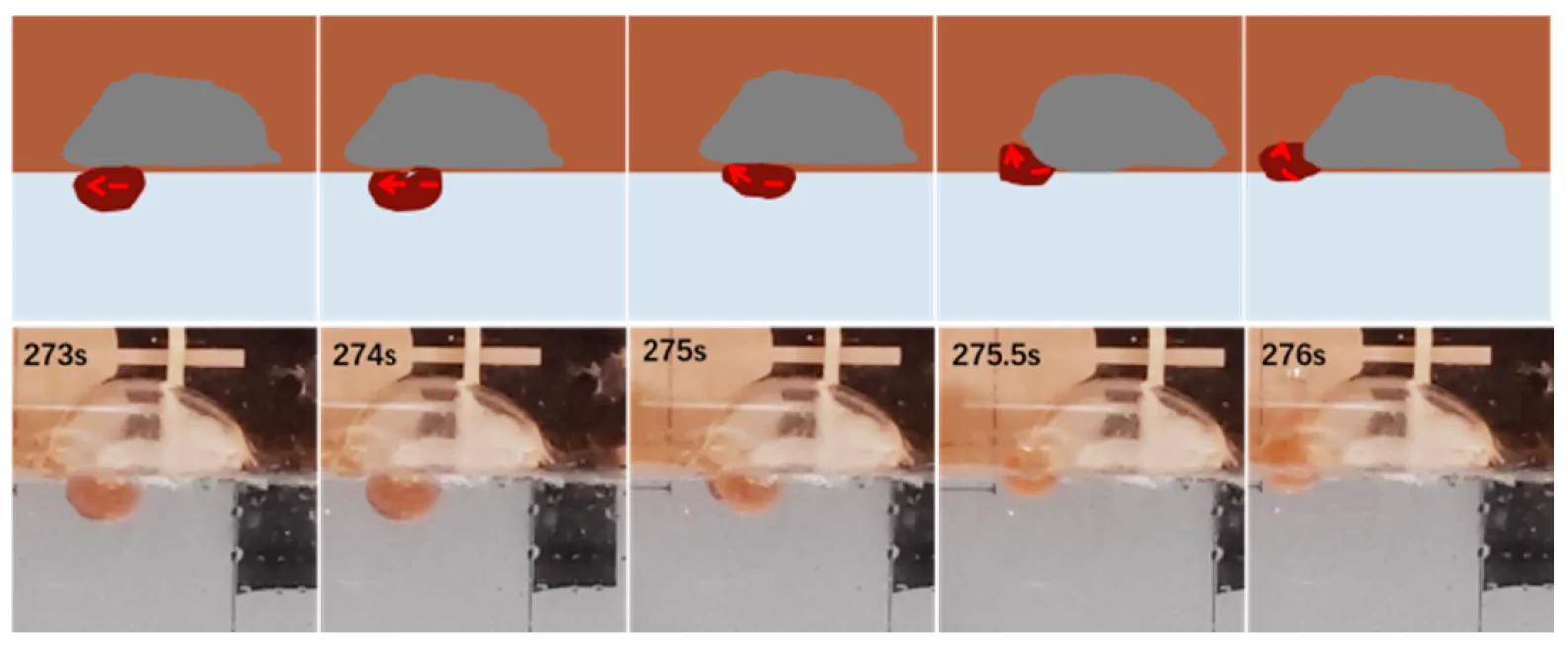
Front view of oil droplet release process under the F-2 condition (second release).

As shown in Figure 21 and Figure 22, the oil droplet release process occurs as follows. As the ice ball on the left melts, the oil droplet becomes directly exposed to the water. At 142 s, the oil droplet begins to slide into the oil layer. Between 142 and 153 s, the oil droplet slowly moves toward the edge of the ice ball. At 154 s, a portion of the oil droplet rises while another portion remains at its original position. At 155 s, the rising oil droplet is completely submerged in the oil layer, and the droplet as a whole is elongated. Within the next 1 s, the middle part of the elongated droplet rapidly thins and eventually breaks, causing the two portions of the oil droplet to separate from each other. One portion directly merges into the oil layer, while the other remains at its original position. Figure 22 shows the second oil droplet release process. At 273 s, the melting of the upper ice ball causes the oil layer to gradually approach the oil droplet, and the ice ball at the two-phase interface forms an arc-shaped structure. The oil droplet begins to move toward the edge of the ice ball. Between 273 and 275 s, the oil droplet slowly moves toward the left side, and at 276 s, the oil droplet rises and directly merges into the oil layer.

#### 3.2.3. Melting of the Ice Ball Under the F-3 Condition

**Figure 23:**
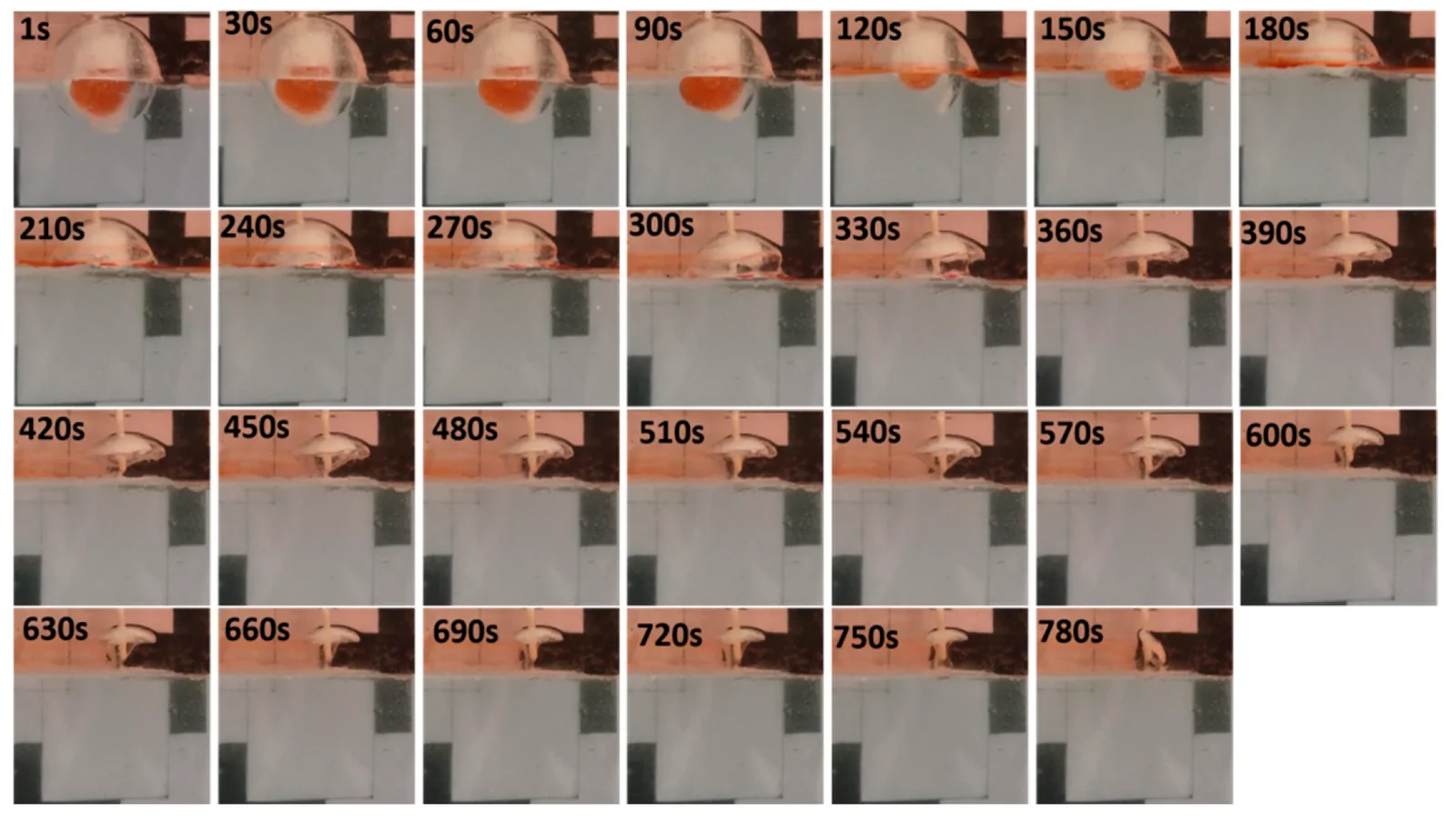
Melting process of the ice ball under the F-3 condition.

As shown in Figure 23, the melting process of the ice ball is presented, with the oil droplet located at the center. Similar to the previous condition F-2, during the first 60 s of melting, the lower ice ball gradually exhibits an inverted triangular shape. At 66 s, a hole forms in the ice ball, exposing the oil droplet to the water. Between 66 and 100 s, the ice layer on the lower left side completely melts. At 101 s, the oil droplet begins to flow outward. After 4 s, a large portion of the oil droplet separates and flows into the oil layer, leaving a relatively small-volume oil droplet at its original position. Similar to the previous condition, this oil droplet does not flow into the oil layer even after the lower ice layer has completely melted at 161 s; instead, it begins to move toward the left side at 166 s and completely merges into the oil layer after 3 s. Subsequently, the upper ice ball exhibits slow and regular melting while maintaining a hemispherical morphology. This process continues from 169 s to 801 s, when the ice ball detaches from the fixed rod, and the ice ball completely melts at 806 s.

**Figure 24:**
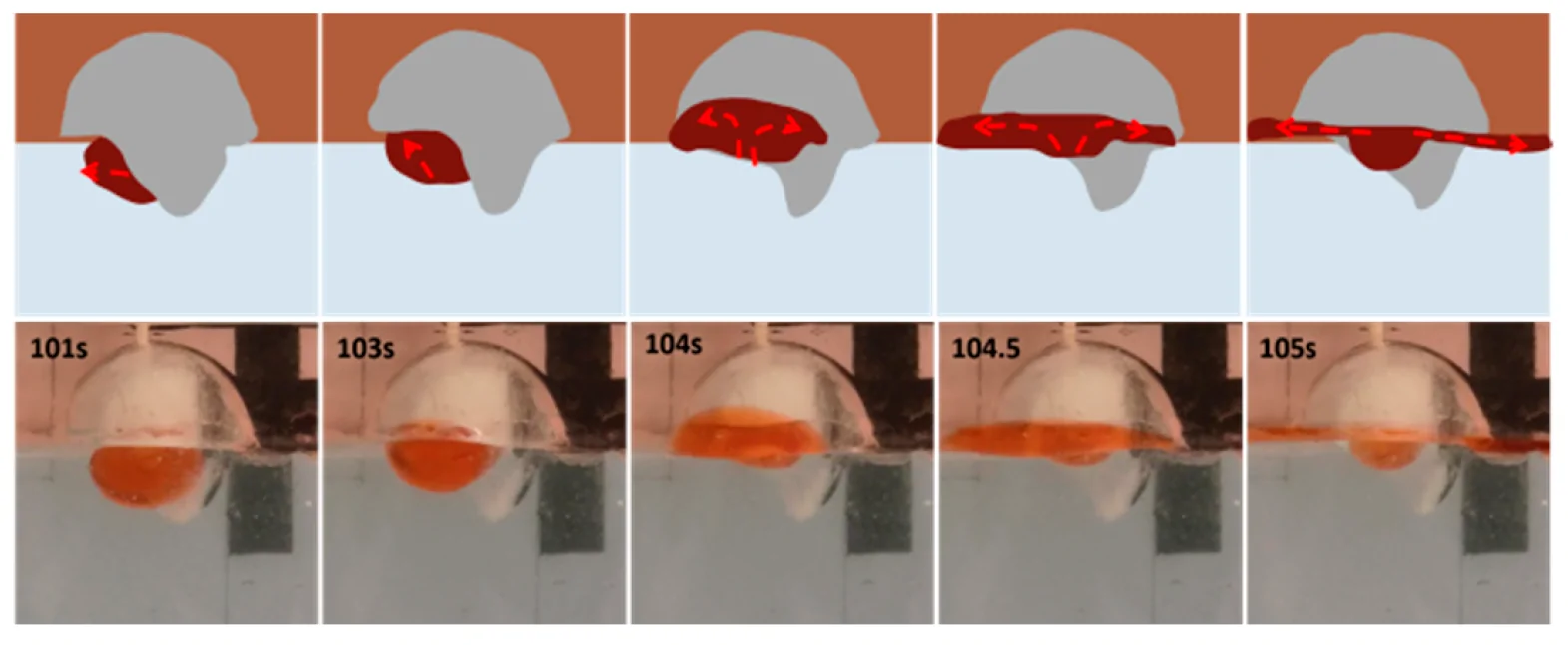
Front view of oil droplet release process under the F-3 condition (first release).

**Figure 25:**
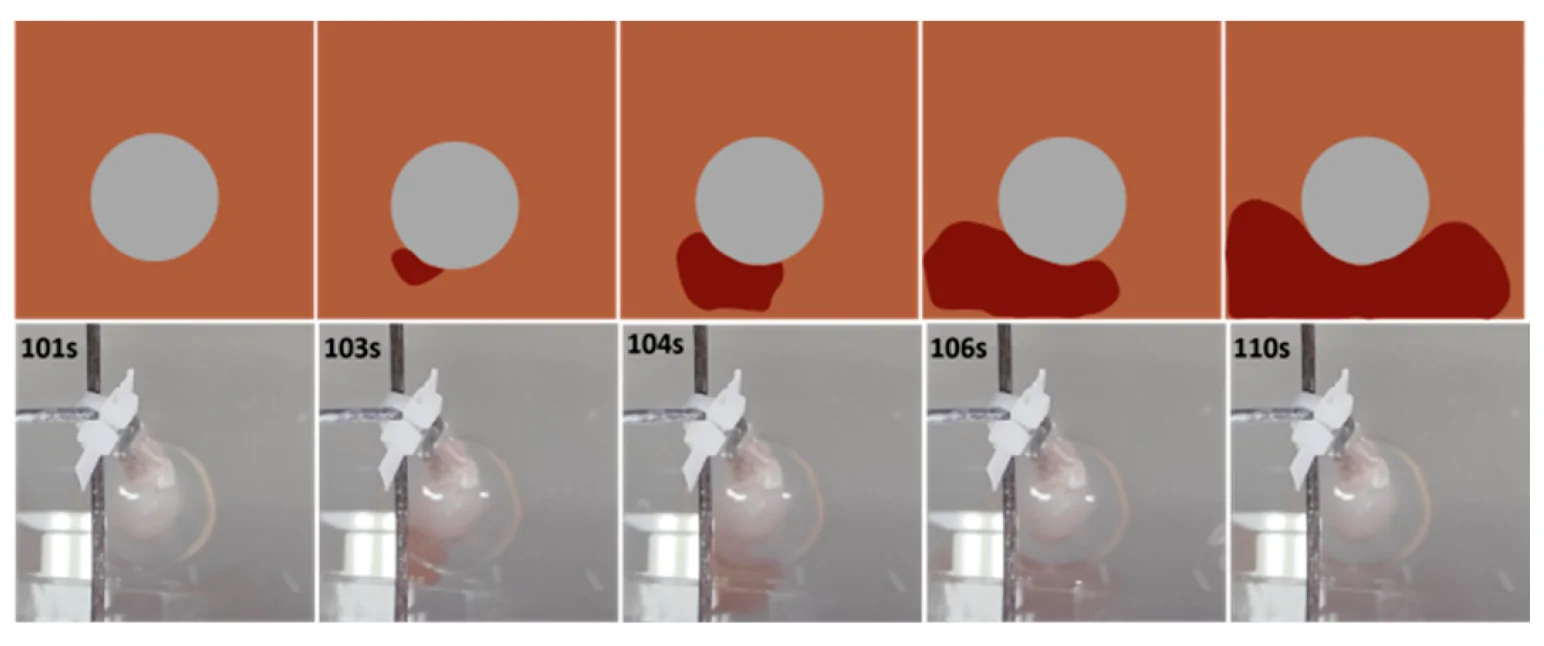
Top view of oil droplet diffusion process under the F-3 condition (first release).

**Figure 26:**
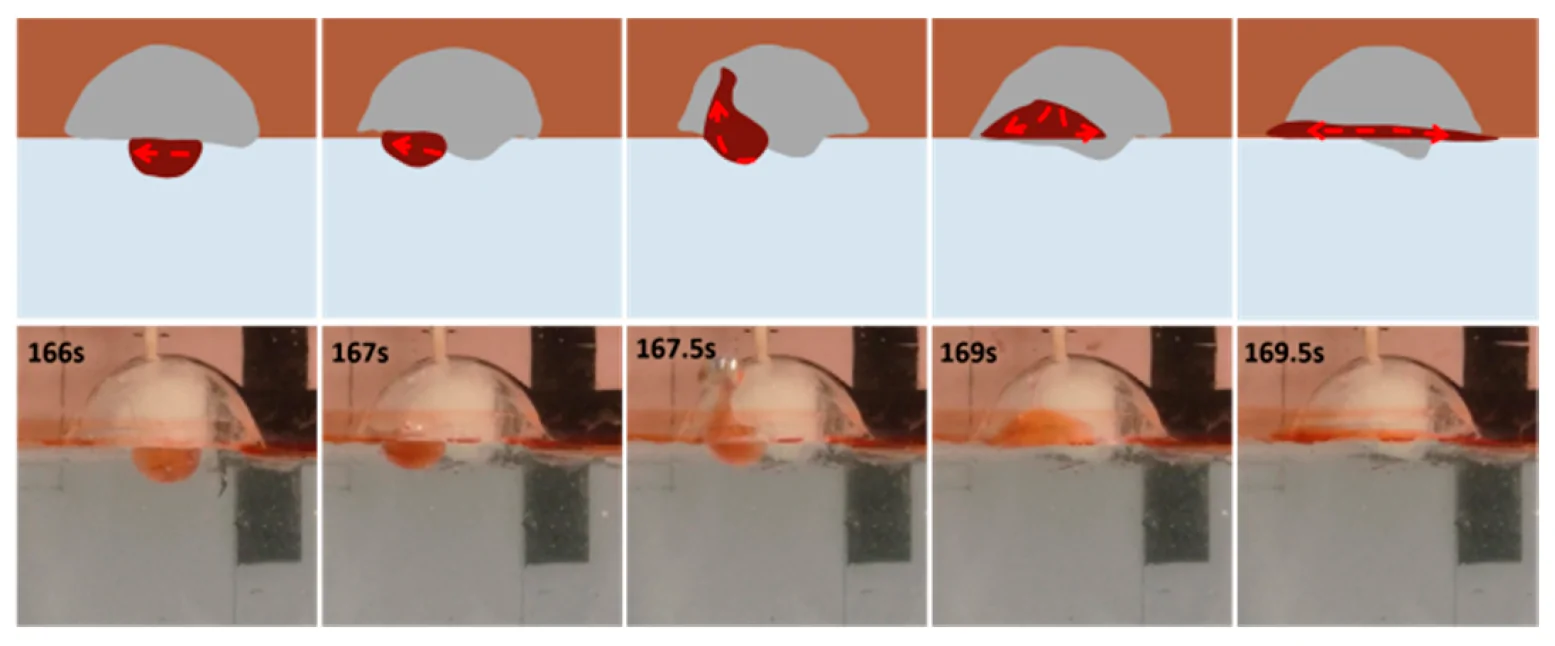
Front view of oil droplet release process under the F-3 condition (second release).

**Figure 27:**
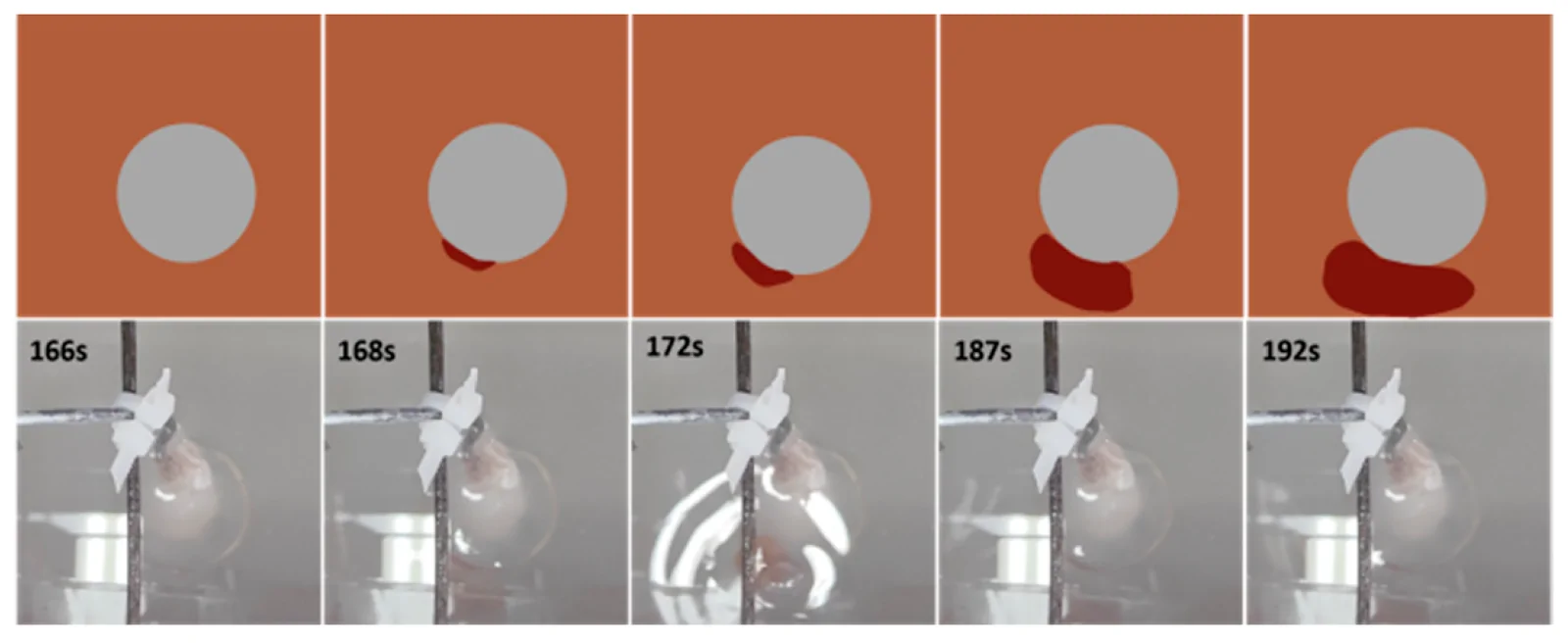
Top view of oil droplet diffusion process under the F-3 condition (second release).

The oil droplet release process under the F-3 condition is shown in Figure 24, Figure 25, Figure 26 and Figure 27. The complete melting of the ice shell on the lower left side of the ice ball causes the oil droplet to become directly exposed to the water. At 101 s, the oil droplet begins to flow outward and moves toward the left side after 1 s. Subsequently, between 102 and 104 s, the oil droplet rises and slides into the oil layer. At 105 s, the oil droplet merges into the oil layer for the first time and spreads toward both sides. From the top view in Figure 25, it can be observed that the oil droplet first flows out from the front of the ice ball, then rapidly spreads along the outflow direction to the edge of the water tank plate, and subsequently spreads toward the surrounding area. It should be noted that in the front view of Figure 24, during 104–105 s, the ice is actually covered by the oil. Subsequently, a portion of the oil droplet with a relatively small mass is observed to remain below the ice ball.

The second sliding of the oil droplet into the oil layer is similar to the second release process under the F-2 condition. At 166 s, the oil droplet slides toward the front edge on the left side of the ice ball for 1 s, i.e., toward the position of the first release. It then immediately rises and slides into the oil layer, and at 169 s, the oil droplet has completely merged into the oil layer, subsequently spreading toward both sides. By observing the second oil droplet spreading process in Figure 27, the droplet rapidly spreads to the edge of the water tank plate in the leakage direction, and then spreads outward in all directions. In both spreading processes of this case, no obvious trend of spreading along the flow direction is observed. Moreover, the oil droplet spreading exhibits a process similar to that in the previous static condition: first, it moves a certain distance due to inertial flow, and then spreads uniformly in all directions.

#### 3.2.4. Melting of the Ice Ball Under the F-4 Condition

**Figure 28:**
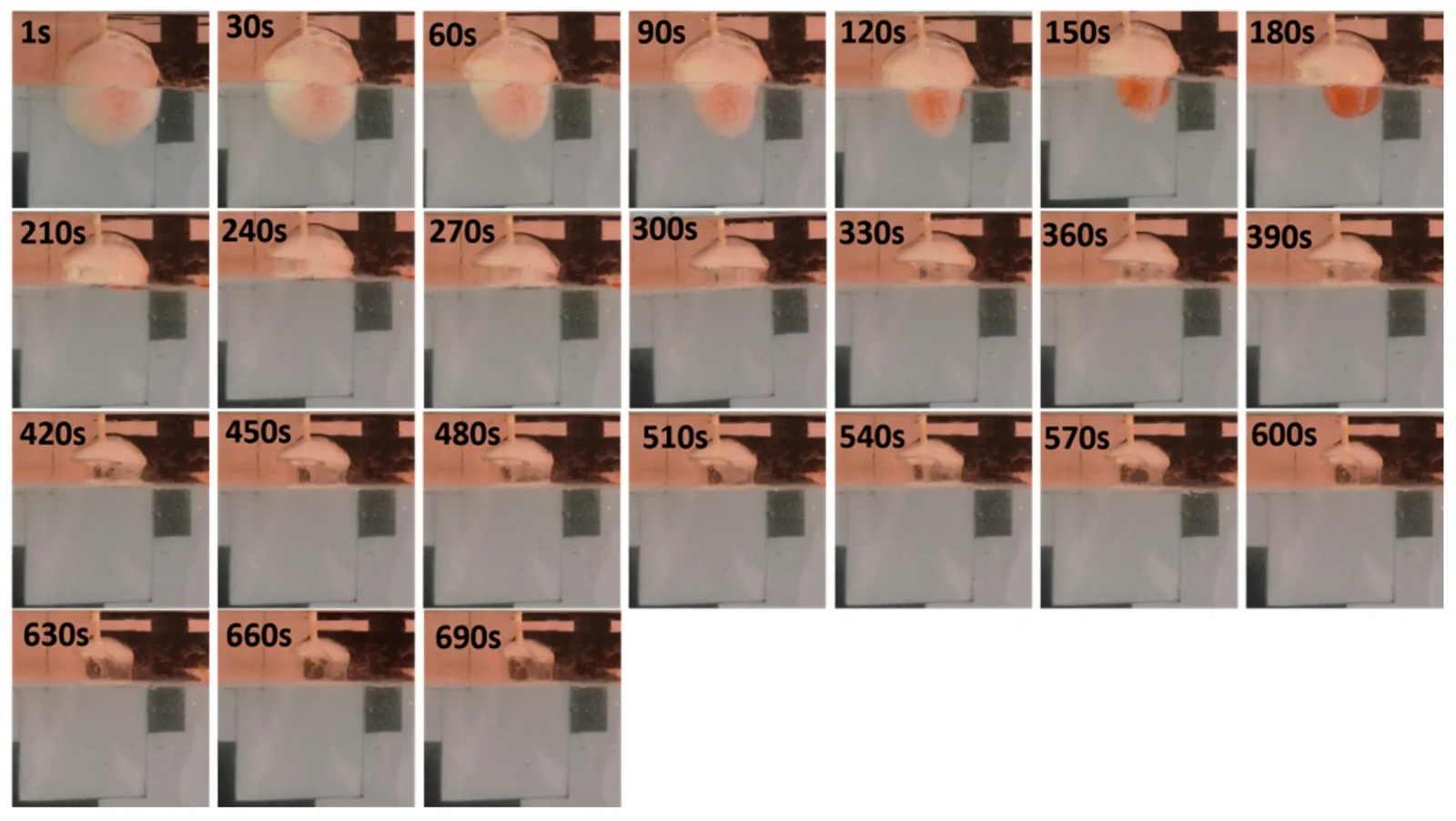
Melting process of the ice ball under the F-4 condition.

Figure 28 shows the melting process of the ice ball under the F-4 condition. During the initial 120 s of melting, the ice ball exhibits a melting process similar to that under the previous conditions, with the upper part gradually thinning at a slow rate and the lower part transitioning from an ellipsoidal shape to an inverted-triangular morphology. At 120 s, a hole forms on the right side of the ice ball, and the oil droplet is exposed to the water for the first time. Between 120 and 180 s, the hole formed in the lower part of the ice ball gradually expands, while the ice shell enclosing the oil droplet gradually decreases in size. Although the oil droplet is directly exposed to the water, it does not flow out and instead remains within a partially enclosed ice shell. The surface tension of the oil droplet delays the release of the oil droplet. At 184 s, as the hole further expands, the oil droplet is driven outward by the parallel flow and begins to flow out, and at 190 s, it has completely merged into the oil layer. This is because, under the impact of the parallel flow, the oil droplet does not flow out from the left side. Meanwhile, the lower right part of the ice ball gradually thins due to the erosion of the flow field and heat conduction of the ice shell, resulting in the formation of a hole. Under the impact of the parallel flow, the oil droplet flows out through the hole on the right side and eventually merges into the oil layer. At 205 s, the lower ice ball has completely melted. The upper ice ball completely melts at 715 s.

**Figure 29:**
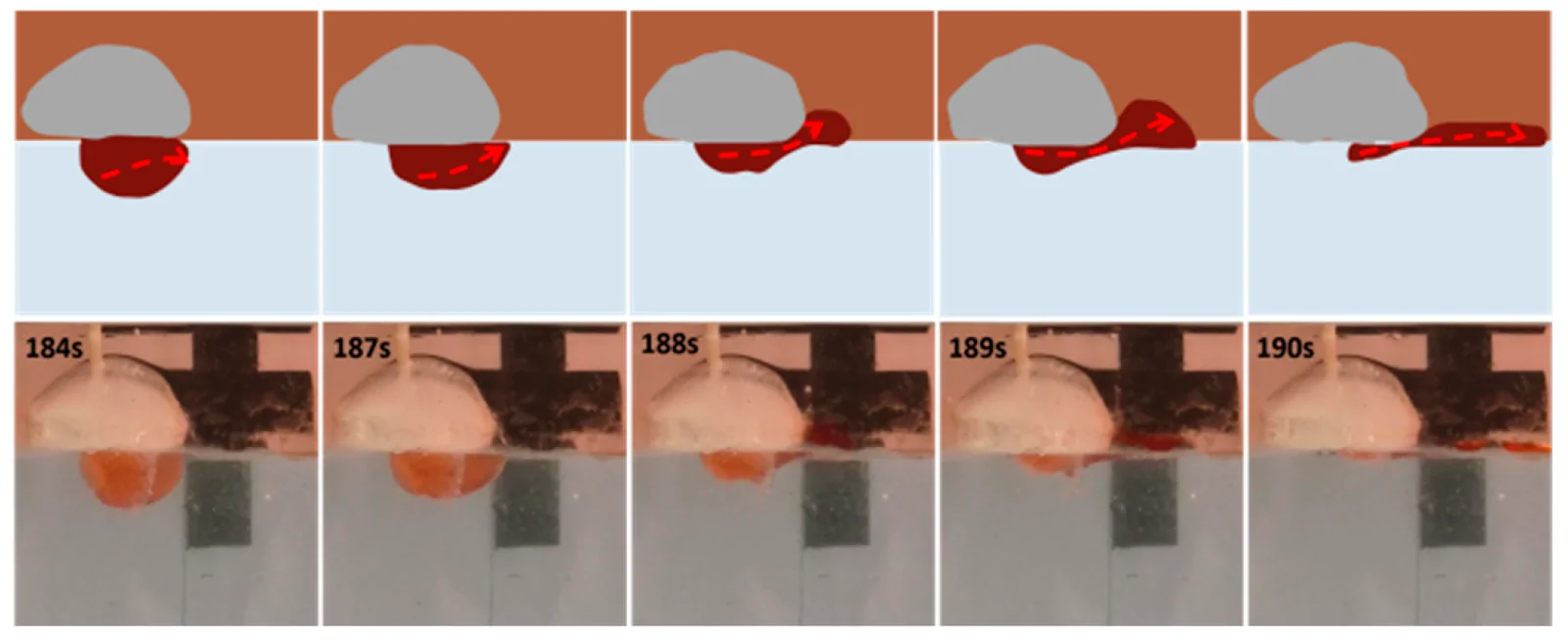
Front view of oil droplet release process under the F-4 condition.

**Figure 30:**
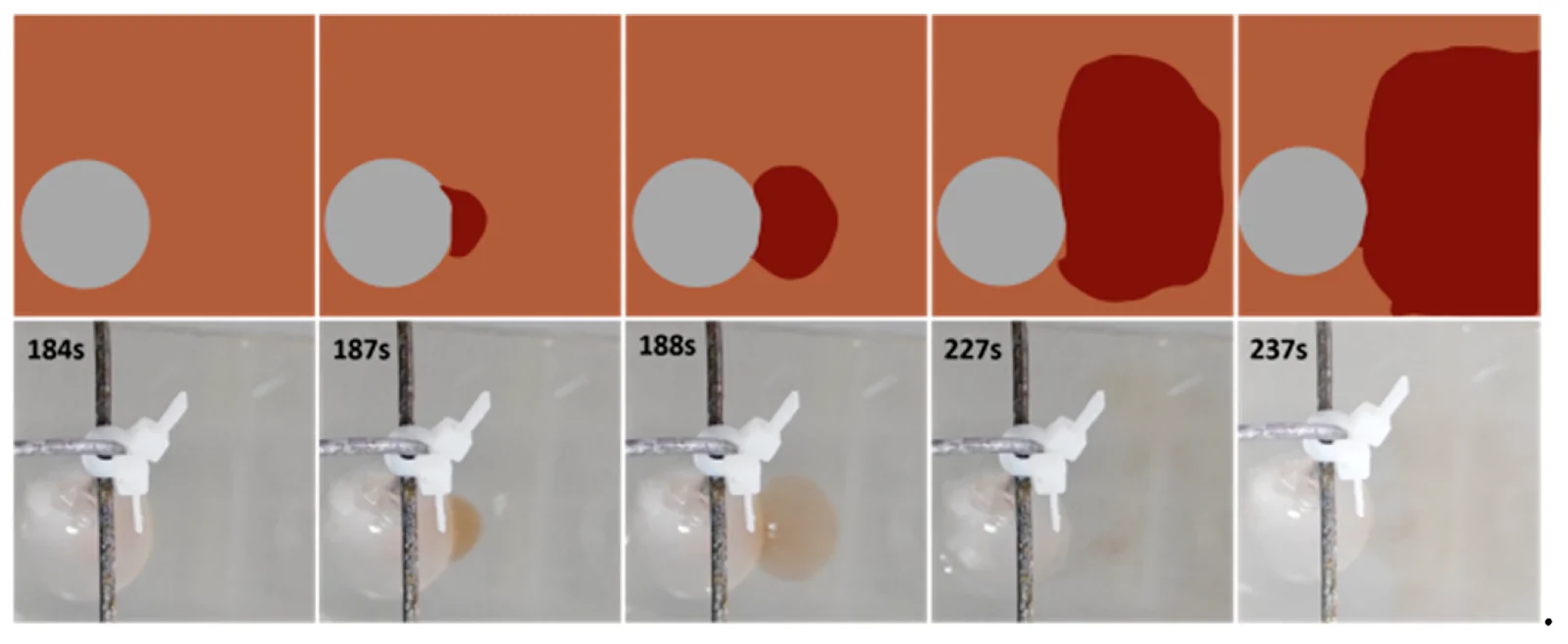
Top view of oil droplet diffusion process under the F-4 condition.

As shown in Figure 29, when the oil droplet is located in the right-side region, the entire oil droplet begins to move toward the hole on the right side between 184 and 187 s. At 188 s, the oil droplet begins to slide into the oil layer and gradually flows out into the oil layer between 188 and 189 s. A portion of the oil droplet remains attached to the edge of the ice ball and connects with the oil droplet that has slid into the oil layer. Finally, at 190 s, the oil droplet has completely merged into the oil layer. From the top view in Figure 30, it can be observed that the oil droplet is driven by the parallel flow to flow into the oil layer from the right side of the ice ball. After merging into the oil layer, the oil droplet similarly spreads for a certain distance in the outflow direction before subsequently spreading outward.

### 3.3. Summary of the Oil Droplet Release Phenomena Under Different Conditions

#### 3.3.1. Effects of Different Factors on the Melting Process

As shown in Table 2, the melting times of the ice balls correspond to the melting times of the upper and lower parts of the ice balls under different conditions. From the melting processes under the S and F series conditions, it can be observed that the melting of the upper ice ball accounts for the majority of the total melting time. Compared with the ice ball submerged in the water below, the melting time is approximately 1.2 to 5 times that of the lower ice ball. The position of the oil droplet has a significant effect on the melting time of the ice ball. When the oil droplet is located in the upper part, the oil droplet occupies the original position of the ice ball and rapidly merges into the oil layer after being exposed. After the oil droplet merges into the oil layer, the heat transfer at the surface of the ice ball is greatly enhanced compared with that of pure ice, resulting in a substantial reduction in the overall melting time. This phenomenon is particularly evident under the S-1 and F-1 conditions, for which the overall melting time is substantially shorter than those under the corresponding conditions in the same series.

The two conditions, static conditions and parallel flow erosion, also have a significant influence on the melting behavior of the ice ball and the oil droplet separation process. When the oil droplet is initially located above the ice ball, the static condition (S-1) and parallel flow condition (F-1) exhibit distinctly different melting and oil droplet release mechanisms. Under the static condition, the overall melting of the ice ball is very slow, and the melting rate in the lower immersed region is much lower than that under parallel flow erosion. The time required for the complete melting of the lower part is approximately twice that under the F-series conditions. Due to this slow melting process, before the lower ice ball has completely melted, the ice shells on both sides enclosing the oil droplet have already melted through, causing the oil droplet to become exposed and merge into the oil layer prematurely, thereby resulting in the separation of the upper and lower ice balls. Under the F-1 condition, however, the erosion of the parallel flow significantly enhances convective heat transfer in the lower part, resulting in a much higher melting rate of the lower ice ball than that under the static condition. Consequently, when the lower ice ball has completely melted, the ice shells on both sides of the oil droplet inside the upper ice ball have not yet melted through. At this point, a hole forms at the bottom of the upper hemispherical ice shell, through which the oil droplet flows into the oil layer. Therefore, the introduction of parallel flow changes the local heat transfer and melting sequence, causing ice balls with identical initial conditions to exhibit completely different oil droplet release paths and morphological evolution characteristics.

#### 3.3.2. Oil Droplet Release Phenomena Under Different Conditions

**Figure 31:**
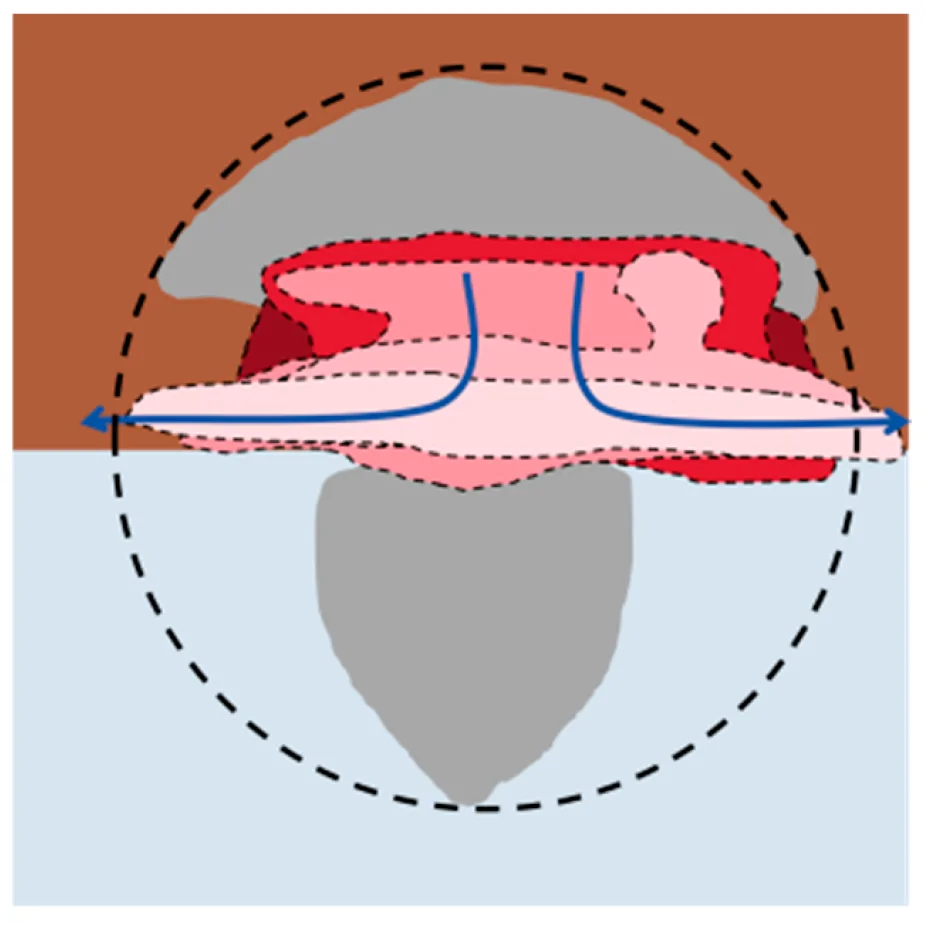
The first type of oil droplet separation process.

The oil droplet release processes at different positions under the two series of conditions can be summarized into five types. Figure 31 shows the first type of oil droplet separation process, which is the phenomenon observed under the S-1 condition. Because the melting of the lower part is relatively slow, the lower ice ball has not completely melted when the oil droplet inside the upper hemisphere flows out. This results in the phenomenon in which the ice shell in the upper part melts, and the oil droplet flows out from around the ice ball and spreads into the oil layer. Figure 31 shows the changes in the oil droplet from the beginning of its outward flow until it completely merges into the oil layer.

**Figure 32:**
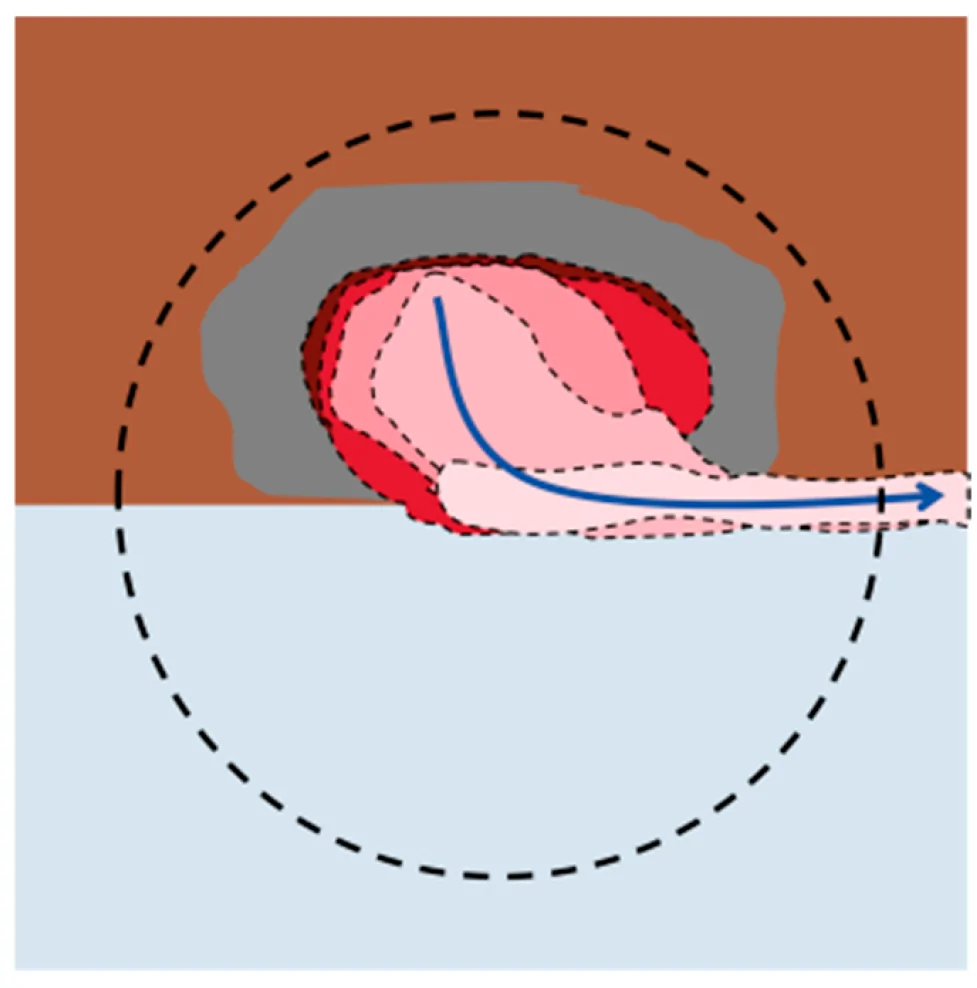
The second type of oil droplet separation process.

The second type is exactly opposite to the first phenomenon. As shown in Figure 32 (F-1 condition as an example), because the lower hemisphere of the ice ball melts relatively quickly, the oil droplet remains enclosed within the upper ice shell. A hole forms as the lower part of the ice shell melts through, and the formation of the hole causes the oil droplet to flow out earlier, resulting in the phenomenon in which the oil droplet flows out from the upper ice shell through the hole on the lower side.

**Figure 33:**
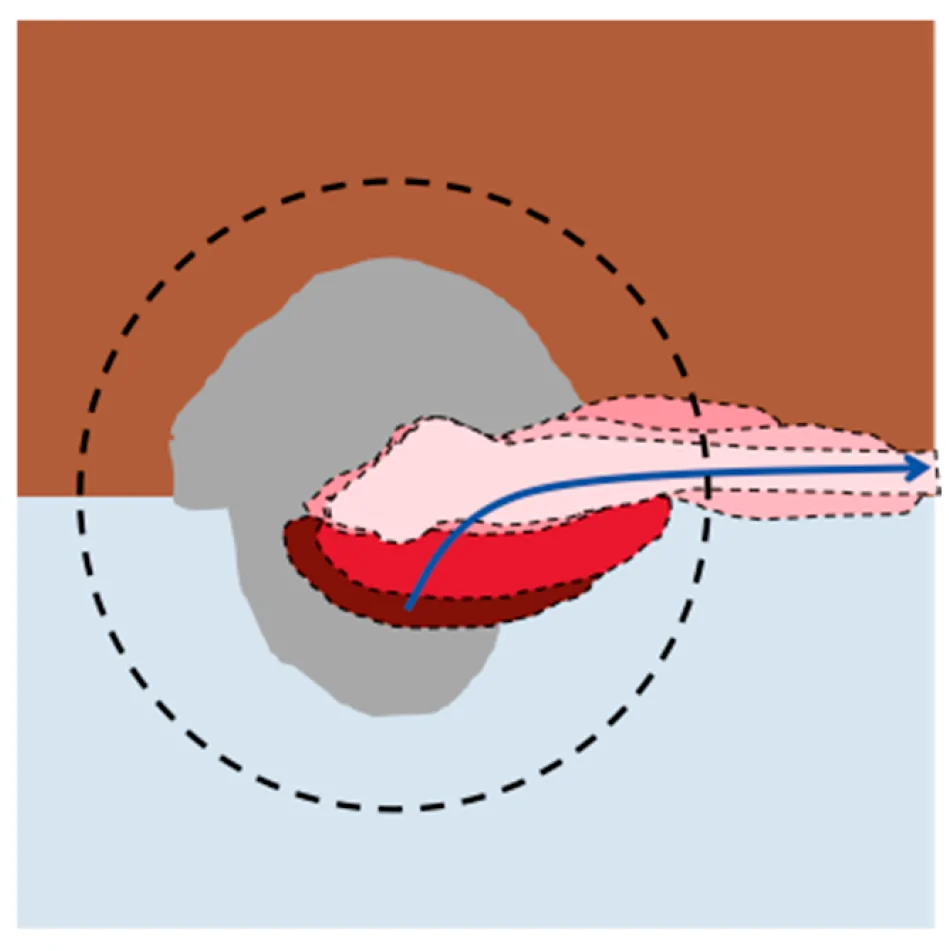
The third type of oil droplet separation process.

The third type corresponds to the case in which the oil droplet is located below the ice ball under static conditions. As shown in Figure 33 (S-3 condition as an example), because the melting rates of the ice on both sides of the lower part are similar, the oil droplet release position is determined by its initial position. The hole generally forms near the thinner side of the ice shell enclosing the oil droplet. After the hole forms, in the absence of the influence of parallel flow, the oil droplet directly flows out through the side where the hole first forms. After flowing out, the oil droplet directly rises and merges into the oil layer without the influence of parallel flow. The schematic diagram in Figure 33 shows the outflow to the right direction, but in reality, there may also be outflow to the left direction or in other directions, such as in the S-2 condition.

**Figure 34:**
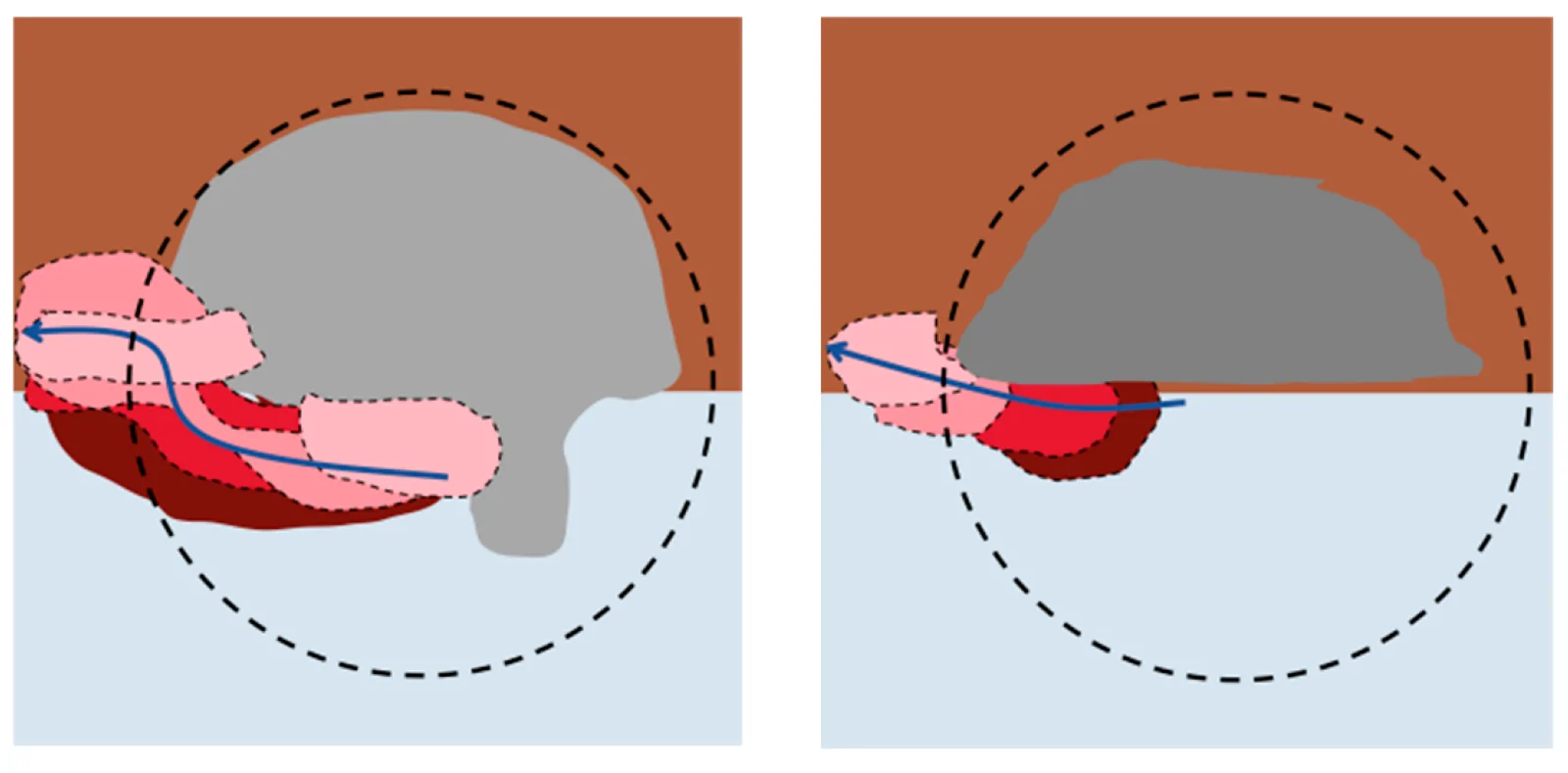
The fourth type of oil droplet separation process (the left panel shows the first separation process, and the right panel shows the second separation process).

The fourth type of behavior was observed in F-2 and F-3, where similar phenomena occurred. As shown in Figure 34, the fourth oil droplet separation process is characterized by a significant two-stage release behavior due to the combined effects of the parallel flow from the left and the interfacial tension of the oil droplet. In the first stage, after the melting of the ice shell exposes a hole, the oil droplet is temporarily constrained by the interfacial confinement effect and the upstream parallel flow, and does not flow out directly. As the ice shell continues to melt and the hole diameter increases, the shear force exerted by the water flow on the oil droplet gradually exceeds the interfacial tension. The main body of the oil droplet is stretched toward the oil layer and slides into it under the flow-induced shear, followed by neck contraction at the connection of the elongated oil droplet, which eventually breaks. The oil droplet with a larger mass directly merges into the oil layer, while a small amount of residual oil droplet contracts into a small spherical shape below the ice ball. As the upper edge of the ice ball gradually melts, the oil layer extends and comes into contact with the residual oil droplet, causing it to move toward the oil layer and eventually merge into it.

**Figure 35:**
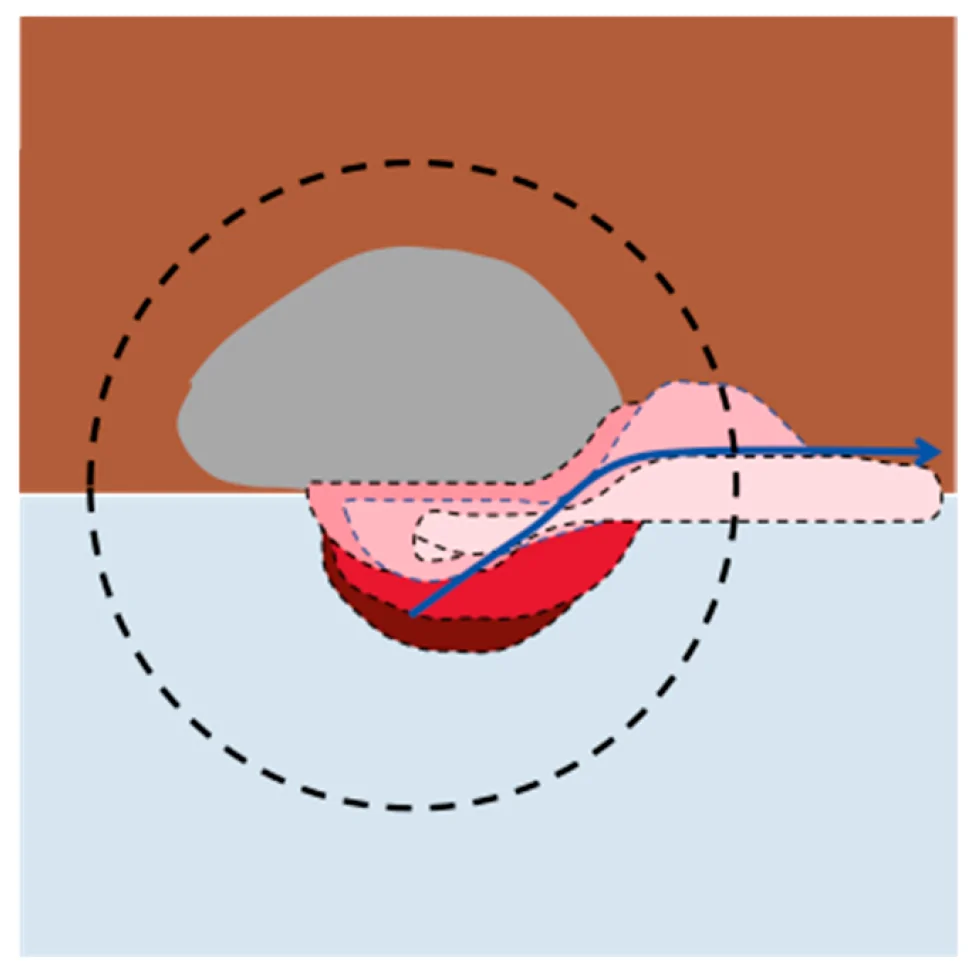
The fifth type of oil droplet separation process.

The fifth type corresponds to the oil droplet located on the right side of the ice ball under the F-4 condition. As shown in Figure 35, its release process exhibits a completely one-time discharge characteristic, which is distinctly different from the staged residual phenomenon observed under the F-2/F-3 conditions. During the early stage of oil droplet release, the oil droplet does not migrate due to the physical barrier of the hole in the ice shell and the interfacial constraint effect. As the ice ball continues to melt, the residual ice shell rapidly becomes thinner, and the hole radius increases significantly, resulting in a substantial reduction in capillary resistance. The shear drag force of the parallel flow becomes absolutely dominant and directly drives the entire oil droplet toward the hole, after which the oil droplet completely merges into the oil layer.

#### 3.3.3. Oil Droplet Separation Time Under Different Conditions

The oil droplet release times under different conditions are listed in Table 3, where the time at which the oil droplet completely flows into the oil layer is recorded. When the oil droplet is initially located above the ice ball, the time required for it to flow into the oil layer is significantly longer than that under the other oil droplet positions within the same series, representing the longest release time among all conditions. Regarding the influence of flow conditions, the release time of the upper oil droplet in the F series (parallel flow) is approximately 2 min shorter than that in the S series (static conditions). Although the flow also accelerates the release process, the release time of the upper oil droplet under the flow conditions remains much longer than that of the oil droplet located in the lower position, indicating that the initial position of the oil droplet exerts a primary influence on the release dynamics. For the conditions involving secondary release, the release time of the second oil droplet is affected by the position of the residual oil droplet, and the interval between the first and second releases varies with the experimental condition. The time interval of the F-2 condition is longer (118 s) than that of the other conditions (about 50–60 s). This may be due to the position of the oil droplet being located in the upstream parallel flow.

## 4. Conclusions and Future Work

### 4.1. Conclusions

Through model simulation experiments, dyed silicone oil was frozen into an ice ball and placed at the slag–metal interface to simulate slag–metal separation. The melting and slag–metal separation phenomena under parallel flow and static conditions were investigated. Based on observations of the melting process, the following conclusions can be drawn:

1. Parallel flow significantly alters the melting process of the ice ball and the oil droplet release behavior. Under static conditions, the oil droplet located above the ice ball flows outward through the holes formed by the melting of the ice shells on both sides, whereas under flowing conditions, the oil droplet flows out through the bottom of the upper hemisphere after the lower part of the ice ball has completely melted.
2. The initial position of the oil droplet and the parallel flow jointly determine the oil droplet separation mode, resulting in five typical separation behaviors: two-sided release, downward release from above the oil–water interface, direct leakage through a single-side hole, two-stage separation from upstream direction, and parallel-flow-driven separation.
3. The diffusion of the dyed oil droplet in the oil layer is only weakly affected by the parallel flow in the water. After flowing out, the oil droplet always moves a certain distance due to inertia and then spreads outward toward the surrounding area.
4. The melting time of the ice ball and the release time of the oil droplet are affected by the initial position of the oil droplet and the parallel flow. When the oil droplet is located in the upper part, the overall melting time of the ice ball is substantially shortened, while the parallel flow greatly reduces the melting time of the lower part of the ice ball. The oil droplet release time in the parallel flow cases is shorter than that in the static conditions. This indicates that flow effectively promotes the separation and release of the oil droplet and substantially shortens the time required for the oil droplet to be released from the ice body.
5. By placing the oil droplets at different positions, the separation phenomena of gangue at different positions within pellets under actual production conditions were simulated. The simulation results show that when the gangue is located in the lower part of the pellet, its separation time is much shorter than when it is located in the upper part. Moreover, when the gangue is located in the lower part of the pellet, the gangue located upstream of the pellet separates faster than that located downstream.

### 4.2. Future Work

The oil droplet release phenomena obtained in this study are mainly limited to the effects of the initial position of the oil droplet and parallel flow under the experimental conditions. Future studies will further investigate the effects of different operating parameters on the melting morphology of the ice ball and the oil droplet release process. By varying key factors such as temperature and flow velocity, the variation characteristics of the ice ball melting rate, melting morphology evolution, and oil droplet release path under different conditions will be analyzed, thereby further clarifying the mechanisms by which these factors affect the oil droplet separation behavior. On this basis, the effects of multiple factors on the melting of the ice ball and oil droplet separation behavior can be further investigated, thereby providing a more comprehensive understanding of the gangue separation behavior during the melting of metallized pellets and providing a reference for the efficient melting of metallized pellets and slag–metal separation in actual smelting processes.

## Figures and Tables

**Table 1:** Experimental scheme for the water model study.

Case	Parallel Flow Velocity/(mm/s)	Oil Droplet Position
S-1	0	Top
S-2	0	Bottom-left
S-3	0	Bottom-middle
S-4	0	Bottom-right
F-1	30	Top
F-2	30	Bottom-left
F-3	30	Bottom-middle
F-4	30	Bottom-right

**Table 2:** Melting time of the ice balls.

Case	Melting Time of the Lower Ice Ball (s)	Overall Melting Time of the Ice Ball (s)
S-1	570	690
S-2	369	740
S-3	352	822
S-4	425	807
F-1	310	514
F-2	251	483
F-3	161	806
F-4	205	715

**Table 3:** Oil droplet release times under different experimental conditions.

Condition	First Release Time (s)	Second Release Time (s)
S-1	422	
S-2	275	
S-3	276	
S-4	179	230
F-1	319	
F-2	158	276
F-3	105	169
F-4	190	

## Data Availability

The original contributions presented in this study are included in the article. Further inquiries can be directed to the corresponding authors.
